# Moderate and severe falls: predictive model based on six years of reports in southern Brazil^*^

**DOI:** 10.1590/1980-220X-REEUSP-2026-0004en

**Published:** 2026-08-31

**Authors:** Sandiely de Araujo Mees, Greici Capellari Fabrizzio, Michelle Mariah Malkiewiez, Lorena Paola Albamonte, Daniela Couto Carvalho Barra, Catiele Raquel Schmidt, Elisiane Lorenzini

**Affiliations:** 1 Universidade Federal de Santa Catarina, Departamento de Enfermagem, Florianópolis, SC, Brazil.; 2 Universidad Nacional de Avellaneda, Departamento de Salud y Actividad Física, Avellaneda, Argentina.

**Keywords:** Patient Safety, Accidental Falls, Hospitalization, Forecasting, Nursing

## Abstract

**Objective:**

To characterize the sociodemographic and clinical profile of patients and identify predictive factors associated with moderate and severe falls.

**Method:**

An analytical cross-sectional study conducted using 300 fall reports (2019–2024). Poisson regression with robust variance was used to estimate prevalence ratios (PRs), logistic regression was performed for predictive modeling, and the area under the receiver operating characteristic (ROC) curve (AUC) was used to assess the model’s discriminatory performance.

**Results:**

Most patients were male (55.0%), with a mean age of 56.4 years and a high prevalence of chronic diseases (86.2%). Most falls resulted in mild harm (54.2%) or moderate harm (44.6%). A hospital stay longer than 14 days (PR = 7.21; 95%CI: 1.68–26.47), male sex (PR = 2.89; 95%CI: 1.15–7.40), falls occurring during the morning shift (PR = 2.54; 95%CI: 1.17–6.73), disorientation (PR = 1.86; 95%CI: 1.11–4.55), chronic diseases (PR = 1.36; 95%CI: 1.11–3.69), and diseases of the circulatory system (PR = 1.58; 95%CI: 1.03–4.31) were associated with a higher risk of moderate/severe falls. Previous hospitalizations were identified as a protective factor (PR = 0.37; 95%CI: 0.22–0.94).

**Conclusion:**

falls were associated with clinical and healthcare-related factors. The model demonstrated moderate discriminatory performance (AUC = 0.77), supporting its potential use in the prevention of moderate and severe falls.

## INTRODUCTION

Hospital falls are among the most frequent adverse events related to patient safety and represent a major global public health concern. According to the World Health Organization, a fall is defined as the unintentional movement of a person to a lower level, which may result in fatal or non-fatal injuries, although most episodes do not result in death^(1)^. Evidence from the Agency for Healthcare Research and Quality indicates that these events are common during hospitalization and, to a large extent, potentially preventable^(2)^.

The Morse Fall Scale is widely recognized as one of the most commonly used tools in clinical practice for assessing the risk of falls among hospitalized patients. It is a validated instrument that is easy to administer and quick to complete, enabling the identification of patients at greater risk of falling through the assessment of multiple risk factors, including previous fall history, mental status, and clinical conditions^(3)^.

The outcomes of falls may be classified as no harm, mild harm, moderate harm, or severe harm. A no-harm event is characterized by the absence of detectable clinical consequences; mild harm involves minor manifestations with minimal functional impairment and requires only monitoring or basic care; moderate harm refers to temporary physical or psychological impairment affecting functional capacity or quality of life; and severe harm involves major consequences, such as severe pain, deformity, or disfigurement, resulting in a substantial reduction in the patient’s autonomy^(1, 2, 3, 4)^.

Hospital falls are influenced by multiple factors, including the physical environment, patients’ clinical conditions, case complexity and acuity, patient engagement and adherence, environmental hazards, and clinical risk screening and assessment, as demonstrated by international studies conducted in Italy and Australia^(5, 6)^. The consequences of falls extend beyond physical and emotional harm, as they may increase the length of hospital stay by an average of 3.9 days in somatic units and 13.2 days in psychiatric units. In addition, they substantially increase healthcare costs, which may reach US$62,521 per episode and US$64,526 when a fall results in injury^(7, 8)^.

In Brazil, falls ranked as the fourth most frequently reported adverse event in the national surveillance system in 2024, with more than 34,000 reports, most of which occurred in hospital settings. Among these events, 9.3% resulted in moderate or severe harm. More than 31,000 of these falls occurred in hospitals. Although Brazil has adopted the Six International Patient Safety Goals, which include fall prevention as recommended by the Joint Commission International, the occurrence of this adverse event remains high^(9)^. During the first half of 2025, the number of reported falls increased substantially, exceeding 80,000 cases, of which more than 76,000 occurred in hospitals^(10)^.

Despite the importance of this topic, gaps remain in the literature regarding the identification of predictive factors associated with the severity of hospital falls in Brazilian healthcare settings, particularly those based on notification data. In this context, predictive models may contribute to the early identification of patients at risk and support the implementation of targeted preventive strategies to improve patient safety^(11)^.

Against this background, this study was guided by the following research questions: What are the sociodemographic and clinical characteristics of patients who experienced falls? Which factors are associated with the occurrence of moderate and severe falls? Therefore, the aim of this study was to analyze reports of hospital falls and identify factors associated with moderate and severe falls in a university hospital in southern Brazil.

## METHOD

### Study Design

This was an analytical cross-sectional study based on the analysis of all fall notifications recorded between 2019 and 2024.

### Study Setting

The study was conducted at a federal university hospital located in Southern Brazil. The institution provides free healthcare through the Unified Health System (SUS - *Sistema* Único *de Saúde*) to patients requiring medium- and high-complexity care.

#### Study Population

A total of 313 records of patients with reported falls were identified. Of these, 13 duplicate records were excluded, resulting in a final study population of 300 patients.

### Data Collection

Data were collected from secondary data sources using records provided by the institution. In addition, electronic medical records were reviewed to obtain clinical variables. Data collection was conducted between June 2024 and February 2025.

The variables initially collected included sociodemographic characteristics (sex, date of birth, race/ethnicity, marital status, and educational attainment); fall notification data (date of notification, work shift, hospital unit, fall risk classification according to the Morse Fall Scale, patient status, location of the incident, and characteristics of the fall); and analytical information (professional category of the reporting professional, possible causes, and risk factors). Subsequently, the Risk Management Team reviewed each report and classified the fall according to the severity of harm (no harm, mild, moderate, severe, or death) and the circumstances of the adverse event. The latter two variables, together with the possible causes and risk factors, were based on the professional’s subjective assessment.

The following clinical variables were subsequently collected: weight, height, smoking status, alcohol consumption, obesity, diabetes mellitus, hypertension, neoplasms, chronic diseases, reason for hospitalization, previous hospitalizations during the previous year (2024), length of hospital stay, and Body Mass Index (BMI).

BMI was calculated as weight divided by height squared and classified according to the World Health Organization criteria, categorizing individuals as underweight, normal weight, overweight, or obese. BMI is a widely used indicator because of its simplicity and applicability and may be useful for identifying nutritional conditions associated with the risk of adverse events, including falls^(12)^.

### Data Analysis and Processing

Data were analyzed using Statistical Package for the Social Sciences (SPSS^®^) version 25.0 (SPSS Inc., Chicago, IL, USA, 2018) for Windows, adopting a 5% significance level. Results were presented using descriptive statistics, including absolute and relative frequencies (n, %), as well as measures of central tendency (mean and median) and variability (standard deviation, range, and interquartile range). The symmetry of continuous variable distributions was assessed using the Kolmogorov–Smirnov test.

For comparisons of proportions, the chi-square goodness-of-fit test was used for categories of a single variable, whereas Cochran’s Q test was applied to dependent variables (multiple-response variables). In the bivariate analysis, Pearson’s chi-square test or Fisher’s exact test was used, as appropriate, together with the estimation of p-value and 95% Confidence Interval (95%CI)^(13, 14)^.

Measures of association were estimated using Poisson regression with robust variance, with calculation of prevalence ratios (PRs) and their corresponding 95%CIs, which constituted the primary inferential approach adopted in this study. Variables with p ≤ 0.250 in the bivariate analysis were considered eligible for inclusion in the multivariable models.

To develop the predictive model and select variables, multivariable logistic regression was performed, considering fall severity classification (moderate/severe vs. mild/no harm) as the dependent variable. Logistic regression was used exclusively for variable selection and assessment of the model’s discriminatory performance and was not used to estimate measures of association.

Model development was conducted in two stages: within-block and between-block analyses. Initially, within-block analyses were performed to identify variables with predictive potential within each group of variables. Subsequently, between-block modeling was conducted by progressively entering the selected variables to develop the final adjusted model. After this stage, the final associations were re-estimated using Poisson regression with robust variance.

The within-block models were constructed according to the following structure: block 1—sociodemographic variables (sex, age, race/ethnicity, marital status, and educational attainment); block 2—clinical characteristics (smoking, alcohol consumption, hypertension, chronic diseases, reason for hospitalization, procedures, and diseases of the circulatory system); block 3—fall-related characteristics (shift, previous hospitalizations, length of hospital stay, and location of the incident); and block 4—possible causes of the incident (impaired balance, disorientation, and the hospital’s physical structure).

The variables “reporting professional category,” “circumstances of the adverse event,” and “risk factors” were excluded from the final model because they showed instability and collinearity, possibly related to selection bias, which compromised the precision of the estimates. The variable “obesity” was also excluded because of the small number of cases in the moderate/severe outcome category (n=9), which precluded its use as a robust predictor in the model.

The Akaike Information Criterion was used to identify the variables included in the final model. Model fit was assessed using the Hosmer–Lemeshow goodness-of-fit test. The final robust model was achieved after 15 steps (likelihood ratio chi-square: initial = 487.156, p = 0.411; final = 622.341, p = 0.564), with no significant differences between the expected and observed models. The discriminatory performance of the final logistic regression model was assessed by calculating the area under the receiver operating characteristic (ROC) curve, which reflects the balance between sensitivity and specificity^(15)^.

In this manuscript, artificial intelligence tools were used exclusively to support linguistic revision and did not influence the data analysis, interpretation of the results, or scientific development of the manuscript.

### Ethical Aspects

The study was approved by the Research Ethics Committee of *Universidade Federal de Santa Catarina* under Certificate of Presentation for Ethical Consideration 75.344723.4.2001.0121 and Opinion 6,712,325.

## RESULTS

A total of 300 patients with reported falls were included in the analysis. The clinical and sociodemographic characteristics of the sample are presented in Table 1. Most were male (55.0%), with a mean age of 56 years, and the 50–59-year age group was the most frequently represented (25.6%). Low prevalences of smoking (39.2%) and alcohol consumption (24.1%) were observed. Among the comorbidities, hypertension (52.1%), diabetes mellitus (39.8%), and neoplasms (35.3%) were the most prevalent, with a high overall prevalence of chronic diseases (86.2%). The main reasons for hospitalization were diseases of the circulatory system (14.0%) and diseases of the respiratory system (10.5%).

**Table 1:** Clinical and sociodemographic characteristics of patients who experienced falls. Measures of central tendency and variability for age – Florianópolis, SC, Brazil, 2026.

Variables	Total (n = 300)^A^		Chi-square goodness-of-fit test p-value
	n	%	
**Sex**			0.083
Female	135	45.0	
Male	165	55.0	
**Age (years)** ^F^			–
Mean ± standard deviation (range)	56.4 ± 17.6 (1 month – 95 years)		
Median (1^st^–3^rd^ quartile)	59 (49 – 69)		
**Diabetes** ^F^	96	39.8	0.002
**Hypertension** ^F^	124	52.1	0.517
**Neoplasm/cancer** ^F^	84	35.3	<0.001
**Chronic diseases** ^F^	219	86.2	<0.001
**Reason for hospitalization** ^A^			–
**Lack of information**	14	4.7	
Presented the information	286	95.3	
**Reason for admission** ^C^			0.008
Procedure	52	18.2	
Diseases of the digestive system	59	20.6	
Respiratory system diseases	30	10.5	
Diseases of the circulatory system	40	14.0	
Diseases of the genitourinary system	14	4.9	
Injuries, poisoning and certain other consequences of external causes	4	1.4	
Symptoms, signs and abnormal clinical and laboratory findings, not elsewhere classified	23	8.0	
Factors influencing health status and contact with healthcare services	11	3.8	
Diseases of the nervous system	13	4.5	
Diseases of the skin and subcutaneous tissue	15	5.2	
Intentionally inflicted injuries	7	2.4	
Pregnancy, childbirth, and the postpartum period	11	3.8	
Diseases of the blood and blood-forming organs and certain immune disorders	4	1.4	
Trauma, injuries, and other complications	4	1.4	
Diseases of the musculoskeletal system and connective tissue	4	1.4	
Endocrine, nutritional and metabolic diseases	3	1.0	
Some infectious and parasitic diseases	2	0.7	
Viral diseases, such as hepatitis, herpes, chickenpox, and coronavirus	1	0.3	

Legend: A – percentage based on the total number of valid cases; B – chi-square goodness-of-fit test (compares response category proportions for the same variable); F – missing data for age (7; 2.3%), (25; 8.3%), diabetes (59; 19.7%), hypertension (62; 20.7%), neoplasia (62; 20.7%), and chronic diseases (46; 15.3%).

Source: Survey data.

Additional sociodemographic variables were also analyzed. Married patients or those living with a partner were more frequent (n = 108; 37.9%) than single patients (n = 94; 33.0%). Regarding educational attainment, incomplete elementary education was the most common level (n = 77; 27.3%), followed by completed high school education (n = 67; 24.4%). With respect to self-reported race/ethnicity, white individuals were more frequent (n = 228; 77.0%) than non-white individuals (n = 68; 23.0%). As for lifestyle habits, 24.0% (n = 54) of patients reported alcohol consumption, and 39.2% (n = 87) were smokers. The mean BMI was 25.2 ± 6.2 kg/m^2^ (range: 13.0–53.1), and the median was 25.2 kg/m^2^ (interquartile range: 21.2–27.8). Overall, 15.0% (n = 33) of patients were classified as obese, considering the high proportion of missing data for this variable (42.0%).

A high proportion of missing data was observed for some of the variables described above, including BMI (42.0%), obesity (28.0%), smoking (26.0%), alcohol consumption (25.3%), marital status (5.0%), and self-reported race/ethnicity (1.3%). All analyses were performed using the available data for each variable.

As shown in Table 2, falls occurred mainly during the morning shift (36.6%) and the night shift (37.0%). Most patients had been classified as high risk for falls before the event (61.7%). Falls occurred more frequently among patients with up to seven days of hospitalization (26.6%). The medical ward accounted for the highest proportion of falls (37.9%), followed by the surgical ward (27.9%) and the adult emergency department (14.6%).

**Table 2:** Absolute and relative distribution of fall notification characteristics – Florianópolis, SC, Brazil, 2026.

Variables	Total (n = 300)^A^		Chi-square goodness-of-fit test p-value^B^
	n	%	
**Year of investigation**			<0.001
2019	62	20.7	
2020	33	11.0	
2021	28	9.3	
2022	44	14.7	
2023	56	18.7	
2024	77	25.7	
**Shift** ^F^			
Morning (7:00 AM – 12:59 PM)	83	36.6	
Afternoon (1:00 PM – 6:59 PM)	60	26.4	
Night (7:00 PM – 6:59 AM)	84	37.0	
**Pre-fall risk assessment (Morse Fal Scale)** ^F^			<0.001
High	121	61.7	
Low	26	13.3	
Moderate	49	25.0	
**Classification of the fall** ^F^			<0.001
Severe	3	1.3	
Moderate	107	44.6	
Mild	130	54.2	
**Previous hospitalizations in the last year (2024)** ^F^			0.143
No	103	55.4	
Yes	83	44.6	
**Hospitalization periods (number of days)** ^D, F^			–
Up to seven days	45	26.6	
From eight to 14 days	41	24.3	
From 15 to 21 days	37	21.9	
From 22 to 28 days	13	7.7	
From 29 to 35 days	15	8.9	
Over 35 days	18	10.7	
**Hospital sector in decline**			<0.001
Surgical clinic	78	27.9	
Medical clinic	106	37.9	
Adult emergency	41	14.6	
Adult Intensive Care Unit	10	3.6	
Pediatrics	4	1.4	
Outpatient clinic	4	1.4	
Rooming-in	11	3.9	
Others	26	9.3	

Legend: A – percentage calculated based on the total number of valid cases; B – chi-square goodness-of-fit test (compares response category proportions for the same variable); D – variable with skewed distribution (Kolmogorov-Smirnov test; p < 0.05); F – missing data for shift (73; 24.3%), risk classification prior to the fall (104; 34.7%), fall classification (60; 20.0%), previous hospitalizations (114; 38.0%), and days of hospitalization (131; 43.7%).

Source: Survey data.

As shown in Table 3, falls occurred more frequently among hospitalized patients (89.2%), predominantly in patient rooms (42.9%) and bathrooms (34.5%), with falls from standing height being the most common mechanism (54.9%).

**Table 3:** Absolute and relative distribution of variables related to the event of falls – Florianópolis, SC, Brazil, 2026.

Variables	Total (n = 300)^A^		Chi-square goodness-of-fit test p-value^B^
	n	%	
**Situation** ^F^			<0.001
Outpatient clinic	5	1.7	
Urgent/emergency care	20	6.7	
Inpatient care	265	89.2	
Other	7	2.4	
**Location of the incident** ^F^			<0.001
Bathroom	102	34.5	
Bedroom	127	42.9	
Hallway	25	8.4	
Other	38	12.8	
Do not know	4	1.4	
**Characteristic** ^F^			<0.001
Shower chair	15	5.1	
Hospital bed	63	21.5	
Stretchers	15	5.1	
Adjustable height	161	54.9	
Wheelchair	9	3.1	
Armchairs	6	2.0	
Other	24	8.2	
**Risk factors**			<0.001^E^
Age	136	45.3	
Diagnosis	182	60.7	
Medications	50	16.7	
Cognitive factors	65	21.7	
Surgery/sedation/anesthesia	26	8.7	
Other	64	21.3	

Legend: A – percentage calculated based on the total number of valid cases; B – chi-square goodness-of-fit test (compares response category proportions for the same variable); E – Cochran’s Q test; F – missing data for situation (3; 1.0%), incident location (4; 1.3%), and characteristic (7; 2.3%).

Source: Survey data.

Among the possible causes, the most frequently identified were impaired balance (n = 111; 37.0%), muscle weakness (n = 93; 31.0%), and disorientation (n = 89; 30.0%). Only 14.0% (n = 43) of the reports identified the hospital’s physical structure and equipment as possible causes of falls. Most reports were submitted by nursing professionals (n = 259; 86.6%). The circumstances most frequently associated with falls by the reporting professionals included the patient’s clinical condition (n = 198; 66.0%), inadequate nutrition (n = 144; 48.0%), and undergoing multiple procedures (n = 126; 42.0%). In contrast, insufficient guidance on fall prevention (n = 61; 20.3%) and staffing levels (n = 31; 10.3%) were less frequently identified as circumstances related to the adverse event.

In the bivariate analysis, the occurrence of moderate/severe falls was associated with male sex (PR = 1.43; 95%CI: 0.94–2.43; p = 0.069), younger age (PR = 0.99; 95%CI: 0.97–1.00; p = 0.093), self-reported non-white race/ethnicity (PR = 1.28; 95%CI: 0.91–1.32; p = 0.086), not living with a partner (PR = 1.66; 95%CI: 0.85–2.77; p = 0.091), and lower educational attainment. Among the clinical and fall-related variables, impaired balance (PR = 0.80; 95%CI: 0.63–1.01; p = 0.064), disorientation (PR = 1.33; 95%CI: 0.86–2.33; p = 0.214), and structural factors (PR = 1.64; 95%CI: 0.83–3.45; p = 0.187) were noteworthy. A statistically significant association was observed only for previous hospitalizations (PR = 0.72; 95%CI: 0.43–0.91; p = 0.009), which acted as a protective factor.

In the intrablock models, self-reported non-white race/ethnicity (PR = 2.46; 95%CI: 1.12–5.41; p = 0.025) and younger age (PR = 0.99; 95%CI: 0.86–1.01; p = 0.099) remained predictors in the sociodemographic block. Self-reported race/ethnicity and age explained 7.8% of the variance in fall severity (Nagelkerke R^2^ = 0.078), with an accuracy of 63.9%. In the clinical block, alcohol consumption (PR = 1.55; 95%CI: 0.92–3.26; p = 0.064), chronic diseases (PR = 1.76; 95%CI: 1.10–3.88; p = 0.004), undergoing a medical procedure (PR = 1.35; 95%CI: 0.95–2.34; p = 0.063), and diseases of the circulatory system (PR = 2.85; 95%CI: 1.19–6.44; p = 0.008) remained in the model, yielding a Nagelkerke R^2^ of 0.156 and an accuracy of 61.7%. In the block related to fall characteristics, the morning shift (PR = 2.35; 95%CI: 1.93–6.98; p = 0.027) and previous hospitalizations (PR = 0.32; 95%CI: 0.41–0.75; p = 0.009) were retained, with a Nagelkerke R^2^ of 0.119 and an accuracy of 60.7%.

As shown in Table 4, there was no substantial reduction in the explained variance (adjusted R^2^: 0.522 vs. 0.497), the strength of the association between variables (0.389 vs. 0.327), or model accuracy (74.4% vs. 69.2%), indicating that the predictive performance was maintained despite the inclusion of fewer variables. Using the backward conditional method, the main predictors of moderate/severe falls were hospital stay longer than 14 days (PR = 7.21; 95%CI: 1.68–26.47), male sex (PR = 2.89; 95%CI: 1.15–7.40), falls occurring during the morning shift (PR = 2.54; 95%CI: 1.17–6.73), disorientation identified as a possible cause of the incident (PR = 1.86; 95%CI: 1.11–4.55), chronic diseases (PR = 1.36; 95%CI: 1.11–3.69), and hospitalization due to diseases of the circulatory system (PR = 1.58; 95%CI: 1.03–4.31), whereas previous hospitalizations remained a protective factor (PR = 0.37; 95%CI: 0.22–0.94) against moderate/severe falls.

**Table 4:** Final poisson regression model for factors associated with the occurrence of moderate/severe falls – Florianópolis, SC, Brazil, 2026.

Independent variables estimated for the final model	p-value	Prevalence ratio for moderate/severe falls		
		PR	95%CI	
**Sociodemographic**				
Male	0.020	2.885	1.146	7.400
**Clinical**				
Diseases of the circulatory system	0.034	1.576	1.026	4.311
Chronic diseases	0.027	1.357	1.105	3.689
**Fall-specific**				
Night shift	0.038	1.000		
Morning shift	0.002	2.541	1.174	6.725
Afternoon shift	0.078	1.118	0.922	3.447
Previous hospitalizations in the last year (2024)	0.026	0.374	0.218	0.941
Hospitalization period				
Up to seven days	0.022			
From eight to 14 days	0.440	3.211	0.166	62.175
From 15 to 21 days and Over 21 days	0.006	7.208	1.680	26.472
**Possible causes of the incident**				
Disorientation	0.017	1.856	1.113	4.551
Constant	0.154	0.006		

Legend: PR – prevalence ratio (estimated by the Poisson regression model); 95%CI – 95% Confidence Interval for the prevalence ratio; model parameters – likelihood-ratio chi-square (622.341; p = 0.564); proportion of explained variance (adjusted R^2^ = 0.497); degree of association between predictor variables and the dependent variable (Cox & Snell = 0.327); confusion matrix (69.2%).

Source: Survey data.

**Figure 1:**
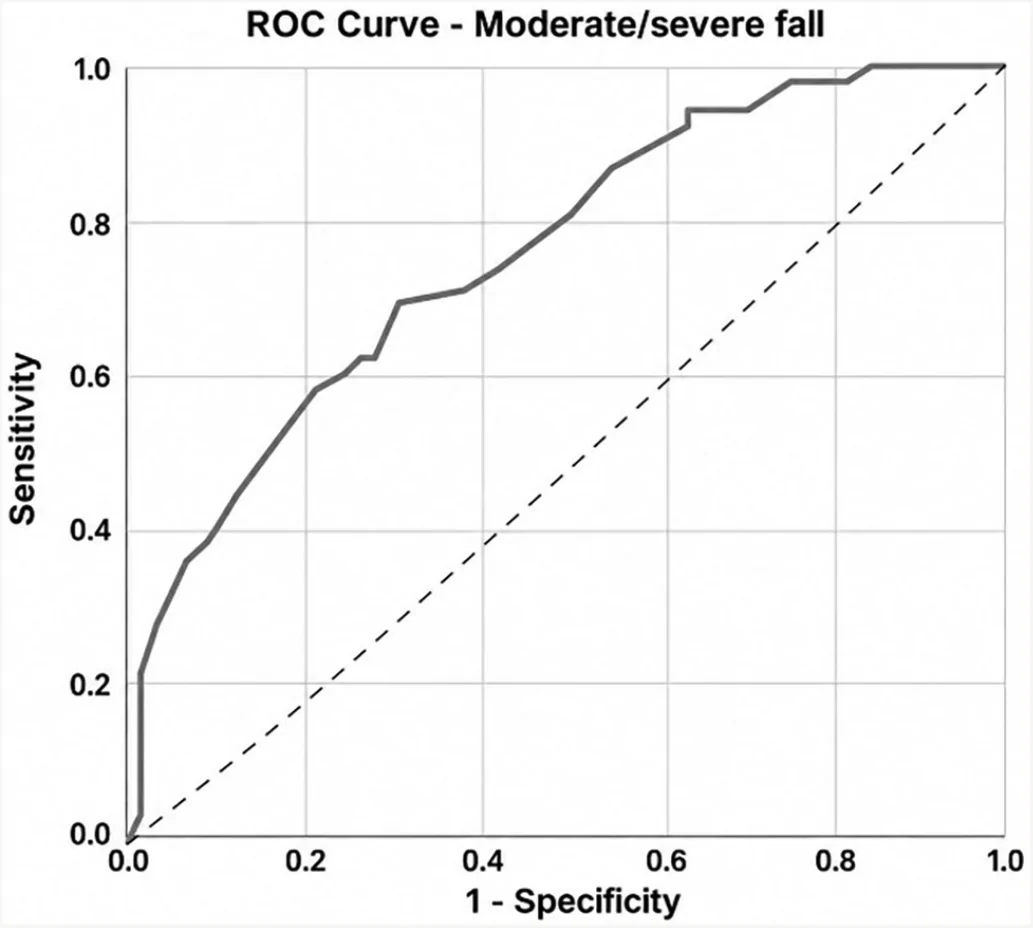
ROC curve for the predictive logistic model for moderate/severe falls - Florianópolis, SC, Brazil, 2026.

Source: Survey data.

Concerning the predictive performance of the final model, the area under the ROC curve was 0.768 (95%CI: 0.681–0.812; p < 0.001) (Figure 1), indicating moderate discriminative ability beyond chance (50.0%). Sensitivity and specificity were 74.6% and 62.3%, respectively. These findings provide evidence that the classifications were not predicted by chance.

## DISCUSSION

The years with the highest concentration of falls were 2024 (25.7%) and 2019 (20.7%). Between these periods, the COVID-19 pandemic occurred, requiring the restructuring of healthcare services and the reallocation of resources, which may have resulted in underreporting of adverse events rather than reflecting a true reduction in the occurrence of falls. Similar findings were reported in a multicenter Italian study, which also demonstrated significant annual variations in the number of in-hospital falls during and after the pandemic^(4)^.

Regarding sex, males predominated (55%) and had an approximately twofold higher risk of moderate and severe falls (PR = 2.89). This finding differs from studies reporting a higher risk among females^(16)^ and may be explained by the hospital characteristics, the patient population served by the healthcare facility, and the other variables included in the study^(16)^.

Chronic diseases were present in 86.2% of the study population and were significantly associated with moderate and severe falls (PR = 1.36), as were diseases of the circulatory system (PR = 1.58). These findings are consistent with evidence linking comorbidities, such as hypertension, diabetes mellitus, heart failure, stroke, and liver disease, to an increased susceptibility to falls and fall-related injuries, particularly among older adults^(17)^. The hospital included in this study provides medium- and high-complexity care within the SUS, concentrating patients with multiple clinical conditions and requiring invasive procedures.

Although falls occurred at similar frequencies during the morning shift (36.6%) and the night shift (37.0%), the predictive model identified the morning shift as being associated with a 2.5-fold higher risk of moderate and severe falls (PR = 2.54). This finding may be related to the greater concentration of nursing care activities during this period, such as bathing, bed linen changes, diagnostic examinations, and patient transportation, as described in international studies^(18)^.

Falls occurred predominantly among hospitalized patients (89.2%), particularly in patient rooms (42.9%) and bathrooms (34.5%). These settings have been identified in the literature as critical locations for fall events. Another study likewise found that falls occurred primarily in patient rooms (64.2%), followed by bathrooms (35.8%) and hallways (10%)^(5)^. These findings highlight the need for structural interventions, such as grab bars, non-slip flooring, and adequate lighting, in addition to enhanced surveillance by the nursing staff.

Hospitalized patients are more susceptible to in-hospital falls due to multiple factors identified by healthcare professionals, including the patient’s clinical condition (66%), inadequate nutrition (48%), advanced age (45.3%), impaired balance (37%), muscle weakness (31%), and clinical diagnosis (60.7%). From this perspective, patients with disorientation identified as a possible cause of the fall were more likely to experience a moderate or severe fall (PR = 1.86). Similar findings have shown that, among other factors, disorientation was associated with a moderate to high risk of falls (OR = 2.40 and 12.54, respectively^(19)^.

Falls from standing height were the predominant mechanism of injury in this study (54.9%) and were associated with the intrinsic factors described above. These findings are consistent with those of a recent systematic review, which identified gait and balance impairments as strong predictors of falls (OR = 2.11). Similarly, disorientation, which was present in 30% of patients in this study and was associated with moderate and severe falls (PR = 1.86), is consistent with international evidence identifying cognitive impairment and delirium as critical risk factors for falls (OR = 2.30)^(20)^.

The most common reason for hospitalization was diseases of the digestive system (20.6%), followed by surgical procedures (18.2%) and diseases of the circulatory system (14.0%). Although diseases of the circulatory system were not prominent in the descriptive analysis, significant prevalence ratios were observed when these variables were included in the predictive model. The most frequently identified conditions in this group were heart failure, peripheral arterial occlusive disease, deep vein thrombosis, stroke, ischemia, and aneurysm. Although the variable referring to patients who underwent procedures was not retained in the final model, amputation was one of the most frequently reported procedures.

In this context, patients with decompensated circulatory diseases may be at increased risk of limb amputation, an event that has a substantial impact on patients’ lives by impairing quality of life, reducing mobility and participation in social activities, and being associated with symptoms of anxiety, depression, and insecurity related to the new circumstances imposed by the condition^(21, 22)^. Furthermore, another study reported a higher prevalence of late falls (occurring more than 10 days after hospitalization) among patients with impairment of the extremities compared with those without such impairment (2.58; 95%CI = 1.40–4.76)^(23)^.

A history of hospitalization during the previous year (2024) was identified as a protective factor against falls resulting in moderate and severe consequences (PR = 0.37). One possible explanation for this finding is that these patients had prior knowledge of fall risk, in addition to greater familiarity with the hospital environment and the preventive measures implemented by the nursing staff. This finding may be further supported by the fact that only 20.3% of the reporting professionals identified a lack of guidance on fall prevention as a circumstance potentially associated with the occurrence of the event^(24)^.

Prolonged hospital stay is strongly associated with poorer clinical outcomes and an increased risk of adverse events. In the present study, a hospital stay longer than 14 days was the factor with the greatest impact on the classification of moderate and severe falls (PR = 7.21). Similar findings were reported by another study, which observed a mean hospital stay of 14.2 days among patients who experienced falls compared with 4.7 days among those who did not, indicating that longer hospitalization is associated with the occurrence of falls^(25)^.

Although prolonged hospital stay is associated with an increased likelihood of falls, it should not be viewed as an isolated risk factor but rather as a reflection of patients’ clinical complexity, which includes greater physical frailty, such as impaired balance and muscle weakness (variables also identified in the present study), as well as greater exposure to medical procedures and the worsening of preexisting conditions. Together, these factors make patients more susceptible to falls resulting in more severe consequences^(26)^.

Furthermore, particularly among older adults, prolonged hospitalization may contribute to hospital-acquired functional decline, including sarcopenia, reduced functional capacity, and impaired postural balance. This deconditioning is associated with an increased risk of falls, fractures, delirium, healthcare-associated infections, loss of independence after discharge, and longer hospital stays. Therefore, preventive measures should be implemented from the time of hospital admission^(27)^.

The predominance of falls among patients classified as being at high risk (61.7%) supports the predictive performance of the Morse Fall Scale. However, this finding suggests that prevention strategies based solely on the total score of the scale may be insufficient, highlighting the need to shift toward interventions tailored to individual risk components, as suggested by a large multicenter study conducted in 72 hospitals. These findings also reinforce the importance of dynamic reassessments, as preventive measures implemented only at admission may fail to reflect changes in the patient’s clinical condition throughout hospitalization^(28)^.

Although only 14% (n = 43) of the reporting professionals identified the hospital’s physical structure and equipment as possible causes of falls, and 10% (n = 31) attributed the event to staffing levels, these factors may be underestimated. Organizational issues tend to be less frequently recognized during adverse event analyses because healthcare professionals generally focus their attention on the patient’s clinical condition and the immediate care processes^(29)^. Nevertheless, even when healthcare professionals are familiar with fall prevention protocols, structural limitations and inadequate staffing may hinder the effective implementation of these preventive measures^(29)^.

The predominance of nursing professionals as the reporting category (86.6%) reflects their central role in patient surveillance and safety due to their continuous proximity to hospitalized patients. However, strengthening multidisciplinary engagement is essential by encouraging all professional categories to participate actively in both the reporting process and the implementation of preventive strategies^(30)^.

This study was conducted in a single general hospital that provides care exclusively through the SUS, reflecting the institutional and healthcare realities of this specific setting. Therefore, the findings should be interpreted with caution regarding their generalizability to other settings, particularly those with different funding models, management structures, or levels of healthcare complexity. Nevertheless, the characteristics of the study hospital may make these findings relevant and potentially applicable to other institutions with similar profiles.

## CONCLUSION

This study characterized patients who experienced in-hospital falls. In this setting, most patients were male, with a mean age of 56 years, and the 50–59-year age group was the most frequently represented. Most patients who experienced falls (86.2%) had chronic diseases. Falls occurred predominantly among hospitalized patients. Among the reasons for hospitalization, diseases of the circulatory and respiratory systems predominated, with most patients having been previously classified as being at high risk for falls. Most falls (54.9%) occurred from standing height.

Furthermore, male sex, longer hospital stay, chronic diseases, diseases of the circulatory system, disorientation, and the morning shift were identified as predictors of moderate and severe falls among hospitalized patients. Having been hospitalized during the previous year was identified as a protective factor against falls with this outcome. The final model demonstrated moderate discriminatory performance, reinforcing its usefulness as a tool to support clinical practice and guide prevention strategies targeted at higher-risk groups.

These findings highlight the importance of investing in targeted preventive measures at the study hospital, such as intensified surveillance during the morning shift, rigorous assessment of the clinical condition of patients with comorbidities, and modifications to the physical environment of patient rooms and bathrooms. This study contributes to advancing patient safety in Brazilian hospitals and underscores the need for future multicenter studies to validate and expand the predictive model proposed herein.

## Data Availability

The entire dataset supporting the results of this study was published in the article itself.
