# Supplementary material for: Moderate and severe falls: predictive model based on six years of reports in southern Brazil
Source: Rev Esc Enferm USP. 2026 Aug 31;60:e20260004. doi: 10.1590/1980-220X-REEUSP-2026-0004en (PMC13537111; doi:10.1590/1980-220X-REEUSP-2026-0004en)
Supplement: Translation

# Translation: Quedas moderadas e graves: modelo preditivo baseado em seis anos de notificações no sul do Brasil^*^

**DOI:** 10.1590/1980-220X-REEUSP-2026-0004pt

**Authors:** Sandiely de Araujo Mees, Greici Capellari Fabrizzio, Michelle Mariah Malkiewiez, Lorena Paola Albamonte, Daniela Couto Carvalho Barra, Catiele Raquel Schmidt, Elisiane Lorenzini

**Affiliations:** 1 Universidade Federal de Santa Catarina, Departamento de Enfermagem, Florianópolis, SC, Brasil.; 2 Universidad Nacional de Avellaneda, Departamento de Salud y Actividad Física, Avellaneda, Argentina.

**Keywords:** Segurança do Paciente, Acidentes por Quedas, Hospitalização, Predição, Enfermagem

## Abstract

**Objetivo:**

Caracterizar o perfil sociodemográfico e clínico dos pacientes e identificar fatores preditivos associados às quedas moderadas e graves.

**Método:**

Estudo transversal analítico com 300 notificações de quedas (2019–2024). Utilizou-se a regressão de Poisson com variância robusta para estimar as razões de prevalência (RP), regressão logística, para análise preditiva, e AUC ROC, para avaliação da capacidade discriminatória do modelo.

**Resultados:**

Predominaram pacientes do sexo masculino (55,0%), com média de 56,4 anos e alta frequência de doenças crônicas (86,2%). As quedas foram majoritariamente leves (54,2%) e moderadas (44,6%). Associaram-se a maior risco de quedas moderadas/graves: tempo de internação >14 dias (RP = 7,21; IC95%:1,68–26,47), sexo masculino (RP = 2,89; IC95%:1,15–7,40), turno da manhã (RP = 2,54; IC95%:1,17–6,73), desorientação (RP = 1,86; IC95%:1,11–4,55), doenças crônicas (RP = 1,36; IC95%:1,11–3,69) e doenças circulatórias (RP = 1,58; IC95%:1,03–4,31) se associaram a maior risco de quedas moderadas/graves. Internações prévias foram fator de proteção (RP = 0,37; IC95%:0,22–0,94).

**Conclusão:**

As quedas associaram-se a fatores clínicos e assistenciais. O modelo apresentou discriminação moderada (AUC = 0,77), com potencial para apoiar a prevenção de quedas com esse desfecho.

## INTRODUÇÃO

As quedas em ambiente hospitalar constituem um dos eventos adversos mais frequentes relacionados à segurança do paciente, representando um problema de saúde pública mundial. Segundo a Organização Mundial da Saúde, a queda é definida como o deslocamento não intencional de uma pessoa para um nível inferior, podendo resultar em lesões fatais ou não fatais, embora a maioria dos episódios não evolua para óbito^(1)^. Evidências da *Agency for Healthcare Research and Quality* indicam que esses eventos são comuns durante a hospitalização e, em grande parte, potencialmente preveníveis^(2)^.

A Escala de Morse é amplamente reconhecida como uma das ferramentas mais utilizadas na prática clínica para a avaliação do risco de quedas em pacientes hospitalizados. Trata-se de um instrumento validado, de fácil aplicação e rápida execução, que permite identificar pacientes com maior probabilidade de sofrer quedas a partir da análise de múltiplos fatores de risco, como histórico prévio, estado mental e condições clínicas^(3)^.

Os desfechos das quedas podem ser classificados como sem dano, dano leve, moderado ou grave. O evento sem dano ocorre sem repercussões clínicas detectáveis; o dano leve envolve manifestações discretas, com mínima perda funcional e necessidade apenas de monitoramento ou cuidados básicos; o dano moderado corresponde a comprometimentos físicos ou psicológicos temporários da capacidade funcional ou da qualidade de vida; e o dano grave envolve repercussões importantes, como dor intensa, deformidades ou desfiguração, com redução substancial da autonomia do paciente^(1,4)^.

As quedas em ambiente hospitalar são influenciadas por múltiplos fatores, incluindo o ambiente físico, as condições clínicas dos pacientes e a complexidade/acuidade dos casos, o envolvimento e a adesão dos pacientes, o perigo/insegurança e a triagem/avaliação de risco clínico, conforme evidenciado em estudos internacionais realizados na Itália e na Austrália^(5,6)^. Os efeitos das quedas vão além das repercussões físicas e emocionais, pois podem aumentar o tempo de internação em, em média, 3,9 dias nas unidades somáticas e 13,2 dias nas psiquiátricas, além de elevar os custos assistenciais, que podem atingir US$62.521 por episódio, chegando a US$64.526 quando há lesão^(7,8)^.

No Brasil, em 2024, as quedas configuraram-se como o quarto evento adverso mais notificado no sistema nacional de vigilância, com mais de 34 mil registros, majoritariamente em ambiente hospitalar. Entre esses, 9,3% resultaram em danos moderados ou graves. A maioria dos episódios (mais de 31 mil) ocorreu em ambiente hospitalar. Embora o país adote as seis Metas Internacionais de Segurança do Paciente, que incluem a prevenção de quedas, conforme preconizado pela *Joint Commission International*, observa-se ainda elevada ocorrência desse evento^(9)^. No primeiro semestre de 2025, observou-se aumento expressivo das notificações, ultrapassando 80 mil casos, dos quais mais de 76 mil ocorreram em hospitais^(10)^.

Apesar da relevância do tema, ainda há lacunas na literatura quanto à identificação de fatores preditivos associados à gravidade das quedas em contextos hospitalares brasileiros, especialmente com base em dados de notificação. Nesse sentido, modelos preditivos podem contribuir para a antecipação de riscos e o direcionamento de estratégias preventivas voltadas à segurança do paciente^(11)^.

Diante desse panorama, este estudo foi norteado pelas seguintes questões: quais são as características sociodemográficas e clínicas dos pacientes acometidos? E quais fatores estão associados à ocorrência de quedas moderadas e graves? Assim, o objetivo foi analisar notificações de quedas hospitalares e identificar fatores associados às quedas moderadas e graves em um hospital universitário do sul do Brasil.

## MÉTODO

### Tipo do Estudo

Trata-se de um estudo transversal analítico, realizado a partir da análise de todas as notificações entre 2019 e 2024.

### Local do Estudo

O estudo foi realizado em um hospital universitário federal situado na região Sul do Brasil. A instituição presta assistência gratuita por meio do Sistema Único de Saúde (SUS) a pacientes de média e alta complexidade.

### População do Estudo

Identificaram-se registros de pacientes com notificação de queda, totalizando 313 casos. Desses, 13 foram excluídos por duplicidade, resultando em uma população de 300 pacientes.

### Coleta de Dados

A coleta de dados foi realizada por meio de fontes secundárias, utilizando-se os registros fornecidos pela instituição. Complementarmente, foram acessados os prontuários eletrônicos e coletadas as variáveis clínicas. Os dados foram coletados entre junho de 2024 e fevereiro de 2025.

As variáveis coletadas inicialmente foram: características sociodemográficas (sexo, data de nascimento, raça, situação conjugal, escolaridade); dados da notificação da queda (data da notificação, turno, setor hospitalar, classificação do risco de queda pela Escala de Morse, situação, local do incidente, características da queda); e informações analíticas (categoria profissional do notificador, possíveis causas, fatores de risco). Posteriormente, a equipe de gestão de risco analisa e classifica a queda quanto ao dano (nenhum, leve, moderado, grave ou óbito) e às circunstâncias do evento adverso. As duas últimas, associadas às possíveis causas e aos fatores de risco, são obtidas por meio da avaliação subjetiva do profissional.

Posteriormente, foram coletadas as seguintes variáveis clínicas: peso; altura; tabagismo; etilismo; obesidade; diabetes; hipertensão arterial sistêmica (HAS); neoplasia; doenças crônicas; motivo da internação; internações prévias no último ano (2024); dias de internação; e Índice de Massa Corporal (IMC).

O IMC é calculado pela razão entre o peso e a altura ao quadrado e classificado conforme os critérios da Organização Mundial da Saúde, permitindo a categorização dos indivíduos em “baixo peso”, “eutrofia”, “sobrepeso” e “obesidade”. Trata-se de um indicador amplamente utilizado por sua simplicidade e aplicabilidade, podendo ser relevante para identificar condições nutricionais associadas ao risco de eventos adversos, incluindo quedas^(12)^.

### Análise e Tratamento de Dados

A análise de dados foi realizada no *Statistical Package for Social Sciences* (SPSS^®^), versão 25.0 (SPSS Inc., Chicago, IL, USA, 2018) para Windows, adotando-se o nível de significância de 5%. Os resultados foram apresentados por estatística descritiva, com distribuições absoluta e relativa (n–%), bem como medidas de tendência central (média e mediana) e de variabilidade (desvio padrão, amplitude e amplitude interquartil). A simetria das distribuições contínuas foi avaliada pelo teste de Kolmogorov-Smirnov.

Para a comparação de proporções, utilizou-se o teste qui-quadrado de aderência para categorias de uma única variável, e o teste de Cochran, para variáveis dependentes (múltiplas respostas). Na análise bivariada, empregaram-se o teste qui-quadrado de Pearson ou o teste exato de Fisher, quando indicado, além da estimativa do valor de p e do Intervalo de Confiança de 95% (IC95%s)^(13,14)^.

As medidas de associação foram estimadas por meio de regressão de Poisson com variância robusta, com cálculo da razão de prevalência (RP) e do respectivo IC95%, sendo essa a abordagem adotada para análise inferencial. Variáveis com p≤0,250 na análise bivariada foram consideradas elegíveis para inclusão nos modelos multivariados.

Para a construção do modelo preditivo e a seleção das variáveis, utilizou-se a regressão logística multivariada, considerando como variável dependente a classificação da queda (moderada/grave vs. leve/sem dano). A regressão logística foi empregada exclusivamente para fins de seleção de variáveis e avaliação da capacidade discriminatória do modelo, não sendo utilizada para estimativa de medidas de associação.

A modelagem foi conduzida em duas etapas: intrabloco e interblocos. Inicialmente, realizou-se a análise intrabloco para identificação das variáveis com potencial preditivo em cada conjunto. Posteriormente, procedeu-se à modelagem interblocos, com inserção progressiva das variáveis selecionadas, visando à construção do modelo final ajustado. Após essa etapa, as associações finais foram reestimadas por meio de regressão de Poisson com variância robusta.

A construção dos modelos intrablocos considerou a seguinte estrutura: bloco 1 – variáveis sociodemográficas (sexo, idade, raça, situação conjugal e escolaridade); bloco 2 – características clínicas (tabagismo, etilismo, HAS, doenças crônicas, motivo da internação, procedimento e doenças do aparelho circulatório); bloco 3 – informações da queda (turno, internações prévias, dias de internação e local do incidente); e bloco 4 – possíveis causas do incidente (falta de equilíbrio, desorientação e estrutura do prédio).

As variáveis “categoria notificadora”, “circunstâncias do evento adverso” e “fatores de risco” foram excluídas da modelagem final por apresentarem instabilidade e colinearidade, possivelmente relacionadas a viés de seleção, o que comprometeu a precisão dos estimadores. A variável “obesidade” também foi excluída devido ao reduzido número de casos na categoria de desfecho moderado/grave (n = 9), o que inviabilizou sua utilização como preditor robusto no modelo.

O Critério de Informação de Akaike possibilitou a seleção das variáveis de interesse para o modelo final. A adequação do modelo de regressão foi avaliada pelo teste de bondade de ajuste do modelo (teste de Hosmer-Lemeshow *goodness-of-fit*). O modelo robusto final foi alcançado em 15 passos (qui-quadrado de razão de verossimilhança: inicial = 487,156; p = 0,411; final = 622,341; p = 0,564), não havendo diferenças significativas entre os modelos esperado e observado. A avaliação da capacidade discriminatória do modelo logístico final alcançado neste estudo foi estimada por meio da análise da área sob a curva ROC. Sua interpretação corresponde ao ajuste entre a especificidade e a sensibilidade^(15)^.

Neste manuscrito, ferramentas de inteligência artificial foram utilizadas exclusivamente para apoio na revisão linguística, sem interferência na análise dos dados, na interpretação dos resultados ou na formulação científica do conteúdo.

### Aspectos Éticos

A pesquisa foi aprovada pelo Comitê de Ética em Pesquisa da Universidade Federal de Santa Catarina, sob Certificado de Apresentação para Apreciação Ética n^o^ 75.344723.4.2001.0121 e Parecer n^o^ 6.712.325.

## RESULTADOS

Foram analisadas as notificações de quedas que ocorreram com 300 pacientes. As características clínicas e sociodemográficas da amostra estão apresentadas na Tabela 1. Predominaram pacientes do sexo masculino (55%), com média de 56 anos, destacando-se a faixa etária de 50 a 59 anos (25,6%). Observou-se uma baixa prevalência de tabagismo (39,2%) e de etilismo (24,1%). Entre as comorbidades, prevaleceram HAS (52,1%), diabetes (39,8%) e neoplasias (35,3%), com predomínio significativo de doenças crônicas (86,2%). Os principais motivos de internação foram doenças do aparelho circulatório (14,0%) e do respiratório (10,5%).

**Tabela 1:** Características clínicas e sociodemográficas de pacientes que sofreram quedas. Medidas de tendência central e de variabilidade para idade – Florianópolis, SC, Brasil, 2026.

Variáveis	Total (n = 300)^A^		Teste X^2^ de aderência Valor de p
	n	%	
**Sexo**			0,083
Feminino	135	45,0	
Masculino	165	55,0	
**Idade (anos)** ^F^			–
Média ± desvio padrão (amplitude)	56,4 ± 17,6 (1 mês – 95 anos)		
Mediana (1º-3º quartil)	59 (49 – 69)		
**Diabetes** ^F^	96	39,8	0,002
**HAS** ^F^	124	52,1	0,517
**Neoplasia/câncer** ^F^	84	35,3	<0,001
**Doenças crônicas** ^F^	219	86,2	<0,001
**Motivo da internação** ^A^			–
**Ausência de informação**	14	4,7	
Apresentou a informação	286	95,3	
**Motivo da internação**			0,008
Procedimento	52	18,2	
Doenças do aparelho digestivo	59	20,6	
Doenças do aparelho respiratório	30	10,5	
Doenças do aparelho circulatório	40	14,0	
Doenças do aparelho geniturinário	14	4,9	
Lesões, envenenamentos e algumas outras consequências de causas externas	4	1,4	
Sintomas, sinais e achados anormais de exames clínicos e de laboratório, classificados em outra parte	23	8,0	
Fatores que influenciam o estado de saúde e o contato com os serviços de saúde	11	3,8	
Doenças do sistema nervoso	13	4,5	
Doenças da pele e do tecido subcutâneo	15	5,2	
Lesões provocadas intencionalmente	7	2,4	
Gravidez, parto e puerpério	11	3,8	
Doenças do sangue e dos órgãos hematopoéticos e alguns transtornos imunitários	4	1,4	
Traumatismos, ferimentos e outras complicações	4	1,4	
Doenças do sistema osteomuscular e do tecido conjuntivo	4	1,4	
Doenças endócrinas, nutricionais e metabólicas	3	1,0	
Algumas doenças infecciosas e parasitárias	2	0,7	
Doenças virais, como hepatite, herpes, varicela e coronavírus	1	0,3	

Legenda: HAS – hipertensão arterial sistêmica; A – percentual obtido com base no total de casos válidos; B - teste qui-quadrado de aderência (compara as proporções das categorias de respostas de uma mesma variável); F v dados faltantes em idade (7; 2,3%),, diabetes (59; 19,7%), HAS (62; 20,7%), neoplasia (62; 20,7%) e doenças crônicas (46; 15,3%).

Fonte: Dados da pesquisa.

Variáveis sociodemográficas adicionais também foram analisadas. Houve maior frequência de pacientes casados/em união estável (n = 108; 37,9%) em comparação aos solteiros (n = 94; 33,0%). Em relação à escolaridade, prevaleceram indivíduos com ensino fundamental incompleto (n = 77; 27,3%), seguidos por aqueles com ensino médio completo (n = 67; 24,4%). Quanto à cor autorreferida, houve maior frequência de indivíduos brancos (n = 228; 77,0%) em relação aos não brancos (n = 68; 23,0%). Em relação aos hábitos de vida, 24,0% (n = 54) dos pacientes apresentavam hábito etilista, e 39,2% (n = 87) eram tabagistas. Quanto ao IMC, a média foi de 25,2 ± 6,2 kg/m^2^ (amplitude: 13,0–53,1), e a mediana, de 25,2 kg/m^2^ (intervalo interquartil: 21,2–27,8), sendo que 15,0% (n = 33) dos pacientes foram classificados como obesos, considerando a elevada proporção de dados faltantes para essa variável (42,0%).

Destaca-se a presença de dados faltantes em algumas das variáveis citadas a cima, como IMC (42,0%), obesidade (28,0%), tabagismo (26,0%), etilismo (25,3%), situação conjugal (5,0%) e cor autorreferida (1,3%). As análises foram conduzidas considerando os dados disponíveis para cada variável.

Conforme a Tabela 2, as quedas ocorreram principalmente nos turnos da manhã (36,6%) e da noite (37,0%). Predominou o alto risco para queda antes do evento (61,7%). Houve maior ocorrência entre pacientes com até sete dias de internação (26,6%). O setor de clínica médica concentrou a maior proporção de quedas (37,9%), seguido pela clínica cirúrgica (27,9%) e pelo setor de emergência adulto (14,6%).

**Tabela 2:** Distribuição absoluta e relativa para as características das notificações das quedas – Florianópolis, SC, Brasil, 2026.

Variáveis	Total (n = 300)^A^		Teste X^2^ de aderência Valor de p^B^
	n	%	
**Ano de investigação**			<0,001
2019	62	20,7	
2020	33	11,0	
2021	28	9,3	
2022	44	14,7	
2023	56	18,7	
2024	77	25,7	
**Turno** ^F^			
Manhã (7h–12h59)	83	36,6	
Tarde (13h–18h59)	60	26,4	
Noite (19h–06h59)	84	37,0	
**Classificação de risco antes da queda (Escala de Morse)** ^F^			<0,001
Alto	121	61,7	
Baixo	26	13,3	
Moderado	49	25,0	
**Classificação da queda** ^F^			<0,001
Grave	3	1,3	
Moderada	107	44,6	
Leve	130	54,2	
**Internações prévias no último ano (2024)** ^F^			0,143
Não	103	55,4	
Sim	83	44,6	
**Dias de internação em período** ^D, F^			–
Até sete dias	45	26,6	
De oito a 14 dias	41	24,3	
De 15 a 21 dias	37	21,9	
De 22 a 28 dias	13	7,7	
De 29 a 35 dias	15	8,9	
Acima de 35 dias	18	10,7	
**Setor hospitalar da queda**			<0,001
Clínica cirúrgica	78	27,9	
Clínica médica	106	37,9	
Emergência adulto	41	14,6	
Unidade de Terapia Intensiva Adulto	10	3,6	
Pediatria	4	1,4	
Ambulatório	4	1,4	
Alojamento conjunto	11	3,9	
Outros	26	9,3	

Legenda: A – percentual obtido com base no total de casos válidos; B – teste qui-quadrado de aderência (compara as proporções das categorias de respostas de uma mesma variável); D – variável com distribuição assimétrica (teste de Kolmogorov-Smirnov; p < 0,05); F – dados ausentes em turno (73; 24,3%), classificação de risco antes da queda (104; 34,7%), classificação da queda (60; 20,0%), internações prévias (114; 38,0%) e dias de internação (131; 43,7%).

Fonte: Dados da pesquisa.

Conforme mostrado na Tabela 3, as quedas se mostraram mais frequentes em pacientes internados (89,2%), predominantemente no quarto (42,9%) e no banheiro (34,5%), sendo mais frequentes as quedas da própria altura (54,9%).

**Tabela 3:** Distribuição absoluta e relativa das variáveis relacionadas ao evento quedas – Florianópolis, SC, Brasil, 2026.

Variáveis	Total (n = 300)^A^		Teste X^2^ de aderência Valor de p^B^
	n	%	
**Situação** ^F^			<0,001
Ambulatório	5	1,7	
Atendimento de urgência/emergência	20	6,7	
Internação	265	89,2	
Outras	7	2,4	
**Local do incidente** ^F^			<0,001
Banheiro	102	34,5	
Quarto	127	42,9	
Corredor	25	8,4	
Outros	38	12,8	
Não sabe informar	4	1,4	
**Característica** ^F^			<0,001
Cadeira de banho	15	5,1	
Leito	63	21,5	
Macas	15	5,1	
Própria altura	161	54,9	
Cadeira de Rodas	9	3,1	
Poltronas	6	2,0	
Outros	24	8,2	
**Fatores de risco**			<0,001^E^
Idade	136	45,3	
Diagnostico	182	60,7	
Medicamentos	50	16,7	
Fatores cognitivos	65	21,7	
Cirurgia/sedação/anestesia	26	8,7	
Outros	64	21,3	

Legenda: A – percentual obtido com base no total de casos válidos; B – teste qui-quadrado de aderência (compara as proporções das categorias de respostas de uma mesma variável); E – teste Q de Cochran; F – dados ausentes em situação (3; 1,0%), local do incidente (4; 1,3%) e característica (7; 2,3%).

Fonte: Dados da pesquisa.

Entre as possíveis causas, destacaram-se a falta de equilíbrio (n = 111; 37,0%), a perda de força (n = 93; 31,0%) e a desorientação (n = 89; 30%). Apenas 14% (n = 43) apontaram a estrutura do prédio e os materiais hospitalares como possíveis causas. A maior parte das notificações foi realizada pela equipe de enfermagem (n = 259; 86,6%). As circunstâncias mais frequentemente associadas às quedas pelos profissionais notificadores incluíram a condição clínica do paciente (n = 198; 66,0%), a nutrição inadequada (n = 144; 48,0%) e a realização de múltiplos procedimentos (n = 126; 42,0%). Por outro lado, a falta de orientação para a prevenção de quedas (n = 61; 20,3%) e o dimensionamento da equipe (n = 31; 10,3%) foram menos frequentemente apontados como circunstâncias relacionadas ao evento adverso.

Na análise bivariada, a ocorrência de quedas moderadas/graves esteve associada ao sexo masculino (RP = 1,43; IC95%: 0,94–2,43; p = 0,069), à menor idade (RP = 0,99; IC95%: 0,97–1,00; p = 0,093), à raça não branca (RP = 1,28; IC95%: 0,91–1,32; p = 0,086), a não viver em união estável (RP = 1,66; IC95%: 0,85–2,77; p = 0,091) e à menor escolaridade. Entre as variáveis clínicas e relacionadas à queda, destacaram-se a falta de equilíbrio (RP = 0,80; IC95%: 0,63–1,01; p = 0,064), a desorientação (RP = 1,33; IC95%: 0,86–2,33; p = 0,214) e os fatores estruturais (RP = 1,64; IC95%: 0,83–3,45; p = 0,187). Observou-se associação estatisticamente significativa apenas para internações prévias (RP = 0,72; IC95%: 0,43–0,91; p = 0,009), configurando-se como fator de proteção.

Nos modelos intrablocos, mantiveram-se como preditores no bloco sociodemográfico a cor autorreferida não branca (RP = 2,46; IC95%: 1,12–5,41; p = 0,025) e a idade mais jovem (RP = 0,99; IC95%: 0,86–1,01; p = 0,099), com variância explicada pela cor autodeclarada e pela idade sobre os tipos de gravidade da queda de 7,8% (R^2^ Nagelkerke = 0,078) e acurácia de 63,9%. No bloco clínico, permaneceram o hábito etilista (RP = 1,55; IC95%: 0,92–3,26; p = 0,064), as doenças crônicas (RP = 1,76; IC95%: 1,10–3,88; p = 0,004), a realização de procedimento (RP = 1,35; IC95%: 0,95–2,34; p = 0,063) e as doenças do aparelho circulatório (RP = 2,85; IC95%: 1,19–6,44; p = 0,008), com R^2^ = 0,156 e acurácia de 61,7%. No bloco referente às características da queda, destacaram-se o turno da manhã (RP = 2,35; IC95%: 1,93–6,98; p = 0,027) e as internações prévias (RP = 0,32; IC95%: 0,41–0,75; p = 0,009), com R^2^ = 0,119 e acurácia de 60,7%.

Conforme apresentado na Tabela 4, no modelo final, não houve redução expressiva da variância explicada (R^2^ ajustado: 0,522 vs. 0,497), da associação entre variáveis (0,389 vs. 0,327) ou da acurácia (74,4% vs. 69,2%), indicando a manutenção do poder preditivo com menor número de variáveis. Pelo método *backward conditional*, os principais preditores de quedas moderadas/graves foram o tempo de internação >14 dias (RP = 7,21; IC95%: 1,68–26,47), o sexo masculino (RP = 2,89; IC95%: 1,15–7,40), a ocorrência de queda no turno da manhã (RP = 2,54; IC95%: 1,17–6,73), apresentar desorientação como possível causa do incidente (RP = 1,86; IC95%: 1,11–4,55), as doenças crônicas (RP = 1,36; IC95%: 1,11–3,69) e o motivo da internação ser doenças do aparelho circulatório (RP = 1,58; IC95%: 1,03–4,31), enquanto internações prévias mantiveram efeito protetor (RP = 0,37; IC95%: 0,22–0,94) para queda moderada/grave.

**Tabela 4:** Modelo final de regressão de Poisson para fatores associados à ocorrência de quedas moderadas/graves – Florianópolis, SC, Brasil, 2026.

Variáveis independentes estimadas para o modelo final	Valor de p	Razão de prevalência para queda moderada/grave		
		RP	IC95%	
**Sociodemográficas**				
Sexo masculino	0,020	2,885	1,146	7,400
**Clínicas**				
Doenças do aparelho circulatório	0,034	1,576	1,026	4,311
Doenças crônicas	0,027	1,357	1,105	3,689
**Específicas da queda**				
Turno noite	0,038	1,000		
Turno manhã	0,002	2,541	1,174	6,725
Turno tarde	0,078	1,118	0,922	3,447
Internações prévias no último ano (2024)	0,026	0,374	0,218	0,941
Período de internação				
Até sete dias	0,022			
De oito a 14 dias	0,440	3,211	0,166	62,175
De 15 a 21 dias e acima de 21 dias	0,006	7,208	1,680	26,472
**Possíveis causas do incidente**				
Desorientação	0,017	1,856	1,113	4,551
Constante	0,154	0,006		

Legenda: RP – razão de prevalência (estimada pelo modelo de regressão de Poisson); IC95% – Intervalo de Confiança de 95% para a razão de prevalência; parâmetros do modelo – qui-quadrado de razão de verossimilhança (622,341; p = 0,564); proporção de variância explicada (R^2^ ajustado = 0,497); grau de associação entre as variáveis preditoras sobre a variável dependente (Cox & Snell = 0,327); matriz de confusão (69,2%).

Fonte: Dados da pesquisa.

Quanto à capacidade preditiva do modelo final, a área sob a curva ROC foi de 0,768 (IC95%: 0,681 – 0,812; p < 0,001) (Figura 1), apontando para uma discriminação moderada além do acaso (50,0%). As estimativas de sensibilidade e especificidade foram de 74,6% e 62,3%, respectivamente. Dessa forma, há evidências de que as classificações não foram preditas por acaso.

**Figura 1:** Curva ROC para o modelo logístico preditivo de queda moderada/grave - Florianópolis, SC, Brasil, 2026.

Fonte: Dados da pesquisa.

## DISCUSSÃO

Os anos com maior concentração de quedas foram 2024 (25,7%) e 2019 (20,7%). Entre esses períodos, ocorreu a pandemia de COVID-19, que exigiu a reestruturação dos serviços de saúde e o redirecionamento de recursos, o que possivelmente resultou em subnotificação de eventos adversos sem representar a redução real na ocorrência. Achados semelhantes foram descritos em um estudo multicêntrico italiano, que também evidenciou variações anuais significativas no número de quedas hospitalares durante e após a pandemia^(4)^.

Em relação ao sexo, identificaram-se o predomínio masculino (55%) e o risco aproximadamente duas vezes maior para quedas moderadas e graves (RP = 2,89). Esse resultado diverge de estudos que apontam maior risco no sexo feminino^(16)^, e pode ser explicado pelas características do hospital estudado, da população atendida no serviço de saúde analisado e das demais variáveis presentes no estudo^(16)^.

As doenças crônicas estavam presentes em 86,2% da população e se associaram significativamente a quedas moderadas e graves (RP = 1,36), assim como as doenças do aparelho circulatório (RP = 1,58). Esses resultados são consistentes com evidências que relacionam comorbidades, como HAS, diabetes, insuficiência cardíaca, acidente vascular cerebral e doença hepática, ao aumento da suscetibilidade a quedas e a lesões principalmente em adultos idosos^(17)^. O hospital analisado atende pacientes de média e alta complexidade do SUS, concentrando casos com múltiplas condições clínicas e procedimentos invasivos.

Embora os períodos matutino (36,6%) e noturno (37,0%) tenham apresentado proporções semelhantes de quedas, o modelo preditivo identificou que o turno da manhã esteve associado ao risco 2,5 vezes maior de quedas moderadas e graves (RP = 2,54). Esse achado pode estar relacionado à maior concentração de atividades assistenciais nesse período, como banhos, troca de roupas de cama, exames e transporte de pacientes, conforme descrito em estudos internacionais^(18)^.

As quedas ocorreram predominantemente em pacientes internados (89,2%), com destaque para quartos (42,9%) e banheiros (34,5%). Esses ambientes são descritos como locais críticos na literatura. Outro estudo também evidenciou que as quedas ocorreram principalmente nos quartos de internação (64,2%), seguidos por banheiros (35,8%) e corredores (10%)^(5)^. Esse achado evidencia a necessidade de intervenções estruturais, como barras de apoio, pisos antiderrapantes e iluminação adequada, além de vigilância intensificada pela equipe de enfermagem.

Pacientes internados apresentam maior suscetibilidade a quedas intra-hospitalares em decorrência de múltiplos fatores identificados pelos profissionais de saúde, como a condição clínica do paciente (66%), a nutrição inadequada (48%), a idade avançada (45,3%), a falta de equilíbrio (37%), a perda de força (31%) e o diagnóstico clínico (60,7%). Nessa perspectiva, observou-se que pacientes com desorientação como possível causa da queda apresentaram maior chance de sofrer uma queda moderada e grave (RP = 1,86). Resultados semelhantes evidenciaram que, além de outros fatores, a desorientação foi um fator de risco médio e alto (OR de 2,40 e 12,54, respectivamente) para quedas^(19)^.

A queda da própria altura foi o mecanismo de maior predomínio neste estudo (54,9%), associada a fatores intrínsecos descritos no parágrafo anterior. Esses achados convergem com uma revisão sistemática recente que identificou o comprometimento de marcha e de equilíbrio como forte preditor de quedas (OR = 2,11). De forma semelhante, a desorientação presente em 30% dos pacientes deste estudo, associada a quedas com desfecho moderado e grave (RP = 1,86), apresenta consonância com evidência internacional que aponta prejuízo cognitivo e *delirium* como fatores críticos para queda (OR = 2,30)^(20)^.

O motivo de internação mais frequente foi o grupo das doenças do aparelho digestivo (20,6%), seguido por procedimentos cirúrgicos (18,2%) e doenças circulatórias (14%). Embora as doenças do aparelho circulatório não tenham se destacado na análise descritiva, foram observadas razões de prevalência significativas no conjunto de variáveis incluídas no modelo preditivo. As condições mais frequentemente identificadas nesse grupo foram insuficiência cardíaca, doença arterial obstrutiva periférica, trombose venosa profunda, acidente vascular cerebral, isquemia e aneurisma. Apesar de a variável referente a pacientes submetidos a procedimentos não ter se mantido no modelo final, a amputação se destacou como um dos principais procedimentos registrados.

Nesse cenário, pacientes com doenças circulatórias descompensadas podem apresentar maior risco de amputação de membros, evento que acarreta impacto substancial na vida do indivíduo, comprometendo a qualidade de vida, reduzindo a mobilidade e a participação em atividades sociais, além de estar associado a sintomas de ansiedade, depressão e insegurança diante das novas condições impostas^(21,22)^. Além disso, outro estudo salienta a maior prevalência de quedas tardias (acima de dez dias) em pacientes com comprometimento das extremidades, em comparação àqueles que não apresentam esses sintomas (2,58; IC95% = 1,40–4,76)^(23)^.

O histórico de internação no último ano (2024) demonstrou ser um fator de proteção para quedas com consequências moderadas e graves (RP = 0,37). Uma possível explicação para esse achado é que tais pacientes já possuíam conhecimentos prévios sobre o risco de quedas, além de maior familiaridade com a rotina hospitalar e com as medidas preventivas implementadas pela equipe de enfermagem. Esse achado pode ser reforçado pelo fato de que apenas 20,3% dos profissionais notificadores identificaram a falta de orientação para prevenção de quedas como circunstância potencialmente associada à ocorrência do evento^(24)^.

O tempo de internação hospitalar prolongado está fortemente associado a piores desfechos clínicos e ao maior risco de eventos adversos. No presente estudo, a permanência superior a 14 dias foi o fator de maior impacto para a classificação de quedas moderadas e graves (RP = 7,21). Achados semelhantes foram descritos por outro autor, que observaram médias de 14,2 dias de internação no grupo com quedas e de 4,7 dias no grupo sem quedas, indicando que a maior permanência hospitalar está associada à ocorrência do evento^(25)^.

Embora o tempo de internação prolongado esteja associado ao aumento da chance de queda, não deve ser entendido apenas como um fator isolado, mas como um reflexo da complexidade clínica do paciente, o que inclui maior fragilidade física, como desequilíbrio e perda de força (variáveis também identificadas neste estudo), além da maior exposição a procedimentos e do agravamento de condições preexistentes. Esses aspectos tornam o paciente mais suscetível a quedas com consequências mais graves^(26)^.

Além disso, especialmente em idosos, o prolongamento da hospitalização pode favorecer a perda funcional adquirida durante a internação, incluindo sarcopenia, redução da capacidade funcional e piora do equilíbrio postural. Esse descondicionamento está associado a um maior risco de quedas, fraturas, delírio, infecções hospitalares, perda de independência após a alta e aumento do tempo de internação. Portanto, são necessárias medidas preventivas desde a admissão hospitalar^(27)^.

A incidência de quedas predominantemente em pacientes classificados como alto risco (61,7%) corrobora a capacidade preditiva da Escala de Morse. Entretanto, esse achado demonstra que a estratégia de prevenção baseada apenas na pontuação total da escala pode ser insuficiente, sendo necessária uma transição para intervenções baseadas nos componentes individuais do risco, conforme sugerido por um amplo estudo multicêntrico realizado em 72 hospitais. Esse achado reforça a necessidade de reavaliações dinâmicas, uma vez que as medidas preventivas estáticas implementadas na admissão podem não acompanhar as mudanças clínicas do paciente ao longo da internação^(28)^.

Apesar de apenas 14% (n = 43) dos profissionais notificadores terem apontado a estrutura física e os materiais hospitalares como possíveis causas da queda e 10% (n = 31) terem relacionado o evento ao dimensionamento da equipe, é possível que esses fatores estejam subestimados. Questões organizacionais tendem a ser menos reconhecidas durante a análise do evento adverso, pois a equipe assistencial geralmente concentra sua atenção nas condições clínicas do paciente e nos processos imediatos de cuidado^(29)^. Entretanto, mesmo quando os profissionais conhecem os protocolos de prevenção, as limitações estruturais e as inadequações no dimensionamento de pessoal podem dificultar a implementação efetiva dessas medidas preventivas^(29)^.

A predominância da enfermagem como categoria notificadora (86,6%) reflete o seu papel central na vigilância e na segurança do paciente, devido à proximidade contínua com os internados. Contudo, é fundamental fortalecer o envolvimento multiprofissional, incentivando que todas as categorias participem ativamente do processo de notificação e da implementação de estratégias preventivas^(30)^.

A pesquisa foi conduzida em um único hospital geral que presta atendimento exclusivamente pelo SUS, o que reflete a realidade institucional e assistencial desse contexto específico. Dessa forma, os resultados devem ser interpretados com cautela quanto à sua generalização para outros cenários, especialmente aqueles com diferentes perfis de financiamento, gestão ou complexidade assistencial. Entretanto, as características do hospital estudado podem tornar os achados relevantes e potencialmente aplicáveis a outras instituições com perfil semelhante.

## CONCLUSÃO

O estudo permitiu caracterizar os pacientes acometidos por quedas hospitalares. Neste cenário, houve predomínio do sexo masculino, com média de idade de 56 anos, sendo a faixa etária de 50 a 59 anos a mais representativa. A maior parte dos pacientes que sofreram queda (86,2%) apresentava doenças crônicas. As quedas ocorreram principalmente entre pacientes internados. Entre os motivos de internação, destacaram-se doenças do aparelho circulatório e respiratório, com predomínio de pacientes previamente classificados como de alto risco para quedas. A maioria das quedas (54,9%) foi da própria altura.

Além disso, foram identificados como preditores para quedas moderadas e graves em pacientes hospitalizados o sexo masculino, o maior tempo de internação, as doenças crônicas, as doenças do aparelho circulatório, a desorientação e o turno matutino. Ter internações prévias no último ano foi um fator de proteção para quedas com esse desfecho. O modelo final apresentou capacidade discriminatória moderada, reforçando a sua utilidade como ferramenta para apoiar a prática clínica e orientar as estratégias de prevenção direcionadas a grupos de maior risco.

Os achados ressaltam a importância de investir em medidas preventivas específicas no hospital estudado, como a intensificação da vigilância nos períodos da manhã, a avaliação rigorosa do estado clínico de pacientes com comorbidades e a adaptação do ambiente físico de quartos e banheiros. O estudo contribui para o avanço da segurança do paciente no contexto hospitalar brasileiro e sinaliza a necessidade de novas pesquisas multicêntricas que validem e ampliem o modelo preditivo aqui proposto.

## Data Availability

Todo o conjunto de dados que dá suporte aos resultados deste estudo foi publicado no próprio artigo.

# Translation: Caídas moderadas y graves: un modelo predictivo basado en seis años de casos reportados en el sur de Brasil^*^

**DOI:** 10.1590/1980-220X-REEUSP-2026-0004es

**Authors:** Sandiely de Araujo Mees, Greici Capellari Fabrizzio, Michelle Mariah Malkiewiez, Lorena Paola Albamonte, Daniela Couto Carvalho Barra, Catiele Raquel Schmidt, Elisiane Lorenzini

**Affiliations:** 1 Universidade Federal de Santa Catarina, Departamento de Enfermagem, Florianópolis, SC, Brasil.; 2 Universidad Nacional de Avellaneda, Departamento de Salud y Actividad Física, Avellaneda, Argentina.

**Keywords:** Seguridad del Paciente, Accidentes por Caídas, Hospitalización, Predicción, Enfermería

## Abstract

**Objetivo:**

Caracterizar el perfil sociodemográfico y clínico de los pacientes e identificar factores predictivos asociados a caídas moderadas y graves.

**Método:**

Estudio transversal analítico con 300 notificaciones de caídas (2019-2024). Se utilizó regresión de Poisson con varianza robusta para estimar las razones de prevalencia (RP), regresión logística para el análisis predictivo y AUC ROC para evaluar la capacidad discriminatoria del modelo.

**Resultados:**

Predominaron los pacientes varones (55,0%), con una edad media de 56,4 años y una alta frecuencia de enfermedades crónicas (86,2%). Las caídas fueron mayoritariamente leves (54,2 %) y moderadas (44,6 %). La duración de la estancia >14 días (RP = 7,21; IC95%: 1,68–26,47), el sexo masculino (RP = 2,89; IC95%: 1,15–7,40), el turno de mañana (RP = 2,54; IC95%: 1,17–6,73), la desorientación (RP = 1,86; IC95%: 1,11–4,55), las enfermedades crónicas (RP = 1,36; IC95%: 1,11–3,69) y las enfermedades circulatorias (RP = 1,58; IC95%: 1,03–4,31) se asociaron con un mayor riesgo de caídas moderadas/graves. Las hospitalizaciones previas fueron un factor protector (PR = 0,37; IC95%: 0,22–0,94).

**Conclusión:**

Las caídas se asociaron con factores clínicos y de atención. El modelo mostró una discriminación moderada (AUC = 0,77), con potencial para apoyar la prevención de caídas con este resultado.

## INTRODUCCIÓN

Las caídas en el entorno hospitalario constituyen uno de los eventos adversos más frecuentes relacionados con la seguridad del paciente, lo que representa un problema de salud pública a nivel mundial. Según la Organización Mundial de la Salud, una caída se define como el desplazamiento involuntario de una persona a un nivel inferior, lo que puede provocar lesiones mortales o no mortales, aunque la mayoría de los episodios no terminan en muerte^(1)^. La evidencia de la *Agency for Healthcare Research and Quality* indica que estos eventos son comunes durante la hospitalización y, en gran medida, potencialmente prevenibles^(2)^.

La Escala de Morse es ampliamente reconocida como una de las herramientas más utilizadas en la práctica clínica para evaluar el riesgo de caídas en pacientes hospitalizados. Se trata de un instrumento validado, de fácil aplicación y rápida ejecución, que permite identificar a los pacientes con mayor probabilidad de sufrir caídas a partir del análisis de múltiples factores de riesgo, como antecedentes previos, estado mental y condiciones clínicas^(3)^.

Los resultados de las caídas pueden clasificarse como sin daño, daño leve, moderado o grave. El evento sin daño ocurre sin repercusiones clínicas detectables; el daño leve implica manifestaciones discretas, con una pérdida funcional mínima y solo requiere monitoreo o cuidados básicos; el daño moderado corresponde a alteraciones físicas o psicológicas temporales de la capacidad funcional o de la calidad de vida; y el daño grave implica repercusiones importantes, como dolor intenso, deformidades o desfiguración, con una reducción sustancial de la autonomía del paciente^(1, 2, 3, 4)^.

Las caídas en el entorno hospitalario se ven influidas por múltiples factores, entre ellos el entorno físico, las condiciones clínicas de los pacientes y la complejidad o gravedad de los casos, la participación y la adherencia de los pacientes, el peligro o la inseguridad, y la clasificación o evaluación del riesgo clínico, tal como lo demuestran estudios internacionales realizados en Italia y Australia^(5−6)^. Los efectos de las caídas van más allá de las repercusiones físicas y emocionales, ya que pueden aumentar el tiempo de hospitalización en un promedio de 3,9 días en las unidades somáticas y de 13,2 días en las psiquiátricas, además de elevar los costos de atención, que pueden alcanzar los 62 521 dólares por episodio, llegando a 64 526 dólares cuando hay lesiones^(7,8)^.

En Brasil, en 2024, las caídas se constituyeron como el cuarto evento adverso más notificado en el sistema nacional de vigilancia, con más de 34 mil registros, en su mayoría en el ámbito hospitalario. De estos, el 9,3 % provocó lesiones moderadas o graves. La mayoría de los episodios (más de 31 mil) ocurrió en el ámbito hospitalario. Aunque el país ha adoptado las seis Metas Internacionales de Seguridad del Paciente, que incluyen la prevención de caídas, tal como lo recomienda la *Joint Commission International*, aún se observa una alta incidencia de este evento^(9)^. En el primer semestre de 2025, se observó un aumento significativo de las notificaciones, que superaron los 80 mil casos, de los cuales más de 76 mil ocurrieron en hospitales^(10)^.

A pesar de la relevancia del tema, aún existen lagunas en la literatura en cuanto a la identificación de factores predictivos asociados a la gravedad de las caídas en entornos hospitalarios brasileños, especialmente a partir de datos de notificación. En este sentido, los modelos predictivos pueden contribuir a la anticipación de riesgos y a la orientación de estrategias preventivas enfocadas en la seguridad del paciente^(11)^.

Ante este panorama, este estudio se guió por las siguientes preguntas: ¿cuáles son las características sociodemográficas y clínicas de los pacientes afectados? ¿Y qué factores están asociados a la ocurrencia de caídas moderadas y graves? Así, el objetivo fue analizar las notificaciones de caídas hospitalarias e identificar los factores asociados a las caídas moderadas y graves en un hospital universitario del sur de Brasil.

## MÉTODO

### Tipo de Estudio

Se trata de un estudio transversal analítico, realizado a partir del análisis de todas las notificaciones entre 2019 y 2024.

### Lugar del Estudio

El estudio se llevó a cabo en un hospital universitario federal ubicado en la región sur de Brasil. La institución brinda atención gratuita a través del Sistema Único de Salud (SUS) a pacientes de complejidad media y alta.

### Población del Estudio

Se identificaron registros de pacientes con notificación de caídas, lo que sumó un total de 313 casos. De estos, 13 fueron excluidos por duplicación, lo que dio como resultado una población de 300 pacientes.

### Recolección de Datos

La recolección de datos se llevó a cabo a través de fuentes secundarias, utilizando los registros proporcionados por la institución. De manera complementaria, se consultaron los expedientes médicos electrónicos y se recopilaron las variables clínicas. Los datos se recopilaron entre junio de 2024 y febrero de 2025.

Las variables recopiladas inicialmente fueron: características sociodemográficas (sexo, fecha de nacimiento, raza, estado civil, nivel educativo); datos de la notificación de la caída (fecha de la notificación, turno, sector hospitalario, clasificación del riesgo de caída según la Escala de Morse, situación, lugar del incidente, características de la caída); e información analítica (categoría profesional de la persona que notificó, posibles causas, factores de riesgo).

Posteriormente, el equipo de gestión de riesgos analiza y clasifica la caída según el daño (ninguno, leve, moderado, grave o muerte) y las circunstancias del evento adverso. Estas dos últimas, junto con las posibles causas y los factores de riesgo, se obtienen mediante la evaluación subjetiva del profesional.

Posteriormente, se recopilaron las siguientes variables clínicas: peso; estatura; tabaquismo; consumo de alcohol; obesidad; diabetes; hipertensión arterial sistémica (HAS); neoplasia; enfermedades crónicas; motivo de la hospitalización; hospitalizaciones previas en el último año (2024); días de hospitalización; e Índice de Masa Corporal (IMC).

El IMC se calcula mediante la relación entre el peso y la altura al cuadrado y se clasifica según los criterios de la Organización Mundial de la Salud, lo que permite categorizar a las personas en «bajo peso», «peso normal», «sobrepeso» y «obesidad». Se trata de un indicador ampliamente utilizado por su simplicidad y aplicabilidad, y puede ser relevante para identificar condiciones nutricionales asociadas al riesgo de eventos adversos, incluidas las caídas^(12)^.

### Análisis y Procesamiento de Datos

El análisis de datos se realizó en el *Statistical Package for Social Sciences* (SPSS^®^), versión 25.0 (SPSS Inc., Chicago, IL, EE. UU., 2018) para Windows, adoptando un nivel de significación del 5 %. Los resultados se presentaron mediante estadísticas descriptivas, con distribuciones absolutas y relativas (n–%), así como medidas de tendencia central (promedio y mediana) y de variabilidad (desviación estándar, amplitud y amplitud intercuartílica). La simetría de las distribuciones continuas se evaluó mediante la prueba de Kolmogorov-Smirnov.

Para la comparación de proporciones, se utilizó la prueba de Chi-cuadrado de concordancia para categorías de una sola variable y la prueba de Cochran para variables dependientes (respuestas múltiples). En el análisis bivariado, se empleó la prueba de Chi-cuadrado de Pearson o la prueba exacta de Fisher, cuando correspondía, además de la estimación del valor de p y del intervalo de confianza del 95 % (IC95 %s)^(13,14)^.

Las medidas de asociación se estimaron mediante regresión de Poisson con varianza robusta, calculando la razón de prevalencia (RP) y su IC95%, siendo este el enfoque adoptado para el análisis inferencial. Las variables con p ≤ 0,250 en el análisis bivariado se consideraron elegibles para su inclusión en los modelos multivariados.

Para la construcción del modelo predictivo y la selección de las variables, se utilizó la regresión logística multivariante, considerando como variable dependiente la clasificación de la caída (moderada/grave frente a leve/sin daño). La regresión logística se empleó exclusivamente con fines de selección de variables y evaluación de la capacidad discriminatoria del modelo, sin utilizarse para la estimación de medidas de asociación.

El modelado se llevó a cabo en dos etapas: intrabloque e interbloques. Inicialmente, se realizó el análisis intrabloco para identificar las variables con potencial predictivo en cada conjunto. Posteriormente, se procedió a la modelación interbloques, con la incorporación progresiva de las variables seleccionadas, con el objetivo de construir el modelo final ajustado. Tras esta etapa, las asociaciones finales se reestimaron mediante regresión de Poisson con varianza robusta.

La construcción de los modelos intrabloque consideró la siguiente estructura: bloque 1 – variables sociodemográficas (sexo, edad, raza, estado civil y nivel educativo); bloque 2 – características clínicas (tabaquismo, alcoholismo, HAS, enfermedades crónicas, motivo de la hospitalización, procedimiento y enfermedades del sistema circulatorio); bloque 3: información sobre la caída (turno, hospitalizaciones previas, días de hospitalización y lugar del incidente); y bloque 4: posibles causas del incidente (falta de equilibrio, desorientación y estructura del edificio).

Las variables «categoría de notificación», «circunstancias del evento adverso» y «factores de riesgo» se excluyeron del modelo final por presentar inestabilidad y colinealidad, posiblemente relacionadas con un sesgo de selección, lo que comprometió la precisión de los estimadores. La variable «obesidad» también se excluyó debido al reducido número de casos en la categoría de resultado moderado/grave (n = 9), lo que impidió su uso como predictor robusto en el modelo.

El criterio de información de Akaike permitió seleccionar las variables de interés para el modelo final. El ajuste del modelo de regresión se evaluó mediante la prueba de bondad de ajuste del modelo (prueba de Hosmer-Lemeshow *goodness-of-fit*). El modelo robusto final se obtuvo en 15 pasos (Chi-cuadrado de la razón de verosimilitud: inicial = 487,156; p = 0,411; final = 622,341; p = 0,564), sin que se observaran diferencias significativas entre los modelos esperado y observado. La evaluación de la capacidad discriminatoria del modelo logístico final obtenido en este estudio se estimó mediante el análisis del área bajo la curva ROC. Su interpretación corresponde al equilibrio entre la especificidad y la sensibilidad^(15)^.

En este manuscrito, las herramientas de inteligencia artificial se utilizaron exclusivamente como apoyo en la revisión lingüística, sin interferir en el análisis de los datos, la interpretación de los resultados ni la formulación científica del contenido.

### Aspectos Éticos

La investigación fue aprobada por el Comité de Ética en Investigación de la Universidad Federal de Santa Catarina, mediante el Certificado de Presentación para Evaluación Ética n.º 75.344723.4.2001.0121 y el Dictamen n.º 6.712.325.

## RESULTADOS

Se analizaron las notificaciones de caídas que se produjeron en 300 pacientes. Las características clínicas y sociodemográficas de la muestra se presentan en la Tabla 1. Predominaron los pacientes de sexo masculino (55 %), con una edad promedio de 56 años, destacando el grupo de edad de 50 a 59 años (25,6 %). Se observó una baja prevalencia de tabaquismo (39,2 %) y de alcoholismo (24,1 %). Entre las comorbilidades, predominaron la HAS (52,1 %), la diabetes (39,8 %) y las neoplasias (35,3 %), con un predominio significativo de enfermedades crónicas (86,2 %). Los principales motivos de hospitalización fueron las enfermedades del sistema circulatorio (14,0 %) y del sistema respiratorio (10,5 %).

**Tabla 1:** Características clínicas y sociodemográficas de los pacientes que sufrieron caídas. Medidas de tendencia central y de variabilidad para la edad – Florianópolis, SC, Brasil, 2026.

Variables	Total (n = 300)^A^		Test X^2^ de adherencia Valor de p
	n	%	
**Sexo**			0,083
Femenino	135	45,0	
Masculino	165	55,0	
**Edad (años)** ^F^			–
Media ± desviación estándar (amplitud)	56,4 ± 17,6 (1 mes – 95 años)		
Mediana (1.º-3.º cuartil)	59 (49 – 69)		
**Diabetes** ^F^	96	39,8	0,002
**HAS** ^F^	124	52,1	0,517
**Neoplasia/cáncer** ^F^	84	35,3	<0,001
**Enfermedades crónicas** ^F^	219	86,2	<0,001
**Motivo de la hospitalización** ^A^			–
**Falta de información**	14	4,7	
Información proporcionada	286	95,3	
**Motivo de la hospitalización**			0,008
Procedimiento	52	18,2	
Enfermedades del aparato digestivo	59	20,6	
Enfermedades del aparato respiratorio	30	10,5	
Enfermedades del aparato circulatorio	40	14,0	
Enfermedades del aparato genitourinario	14	4,9	
Lesiones, intoxicaciones y algunas otras consecuencias de causas externas	4	1,4	
Síntomas, signos y hallazgos anormales en exámenes clínicos y de laboratorio, clasificados en otra parte	23	8,0	
Factores que influyen en el estado de salud y el contacto con los servicios de salud	11	3,8	
Enfermedades del sistema nervioso	13	4,5	
Enfermedades de la piel y del tejido subcutáneo	15	5,2	
Lesiones provocadas intencionalmente	7	2,4	
Embarazo, parto y puerperio	11	3,8	
Enfermedades de la sangre y de los órganos hematopoyéticos y algunos trastornos inmunitarios	4	1,4	
Traumatismos, lesiones y otras complicaciones	4	1,4	
Enfermedades del sistema osteomuscular y del tejido conjuntivo	4	1,4	
Enfermedades endocrinas, nutricionales y metabólicas	3	1,0	
Algunas enfermedades infecciosas y parasitarias	2	0,7	
Enfermedades virales, como la hepatitis, el herpes, la varicela y el coronavirus	1	0,3	

Leyenda: HAS – hipertensión arterial sistémica; A – porcentaje obtenido a partir del total de casos válidos; B – prueba de Chi-cuadrado de concordancia (compara las proporciones de las categorías de respuesta de una misma variable); F: datos faltantes en edad (7; 2,3 %), diabetes (59; 19,7 %), HAS (62; 20,7 %), neoplasia (62; 20,7 %) y enfermedades crónicas (46; 15,3 %).

Fuente: Datos de la investigación.

También se analizaron otras variables sociodemográficas. Se observó una mayor frecuencia de pacientes casados o en unión estable (n = 108; 37,9 %) en comparación con los solteros (n = 94; 33,0 %). En cuanto al nivel educativo, predominaron las personas con educación primaria incompleta (n = 77; 27,3 %), seguidas de aquellas con educación secundaria completa (n = 67; 24,4 %). En cuanto al color de piel autodeclarado, hubo una mayor frecuencia de personas blancas (n = 228; 77,0 %) en comparación con las no blancas (n = 68; 23,0 %). En cuanto a los hábitos de vida, el 24,0 % (n = 54) de los pacientes presentaba hábitos de consumo excesivo de alcohol, y el 39,2 % (n = 87) eran fumadores. En cuanto al IMC, la media fue de 25,2 ± 6,2 kg/m^2^ (rango: 13,0–53,1), y la mediana, de 25,2 kg/m^2^ (rango intercuartílico: 21,2–27,8), si bien el 15,0 % (n = 33) de los pacientes se clasificaron como obesos, teniendo en cuenta la elevada proporción de datos faltantes para esta variable (42,0 %).

Cabe destacar la presencia de datos faltantes en algunas de las variables mencionadas anteriormente, como el IMC (42,0 %), la obesidad (28,0 %), el tabaquismo (26,0 %), el consumo de alcohol (25,3 %), el estado civil (5,0 %) y el color de piel autodeclarado (1,3 %). Los análisis se realizaron considerando los datos disponibles para cada variable.

Según la Tabla 2, las caídas ocurrieron principalmente en los turnos de la mañana (36,6 %) y de la noche (37,0 %). Predominó el alto riesgo de caída antes del evento (61,7 %). Hubo una mayor incidencia entre los pacientes con hasta siete días de hospitalización (26,6 %). El sector de medicina interna concentró la mayor proporción de caídas (37,9 %), seguido por el de cirugía (27,9 %) y el de urgencias para adultos (14,6 %).

**Tabla 2:** Distribución absoluta y relativa de las características de las notificaciones de caídas – Florianópolis, SC, Brasil, 2026.

Variables	Total (n = 300)^A^		Test X^2^ de adherencia Valor de p ^B^
	n	%	
**Año de investigación**			<0,001
2019	62	20,7	
2020	33	11,0	
2021	28	9,3	
2022	44	14,7	
2023	56	18,7	
2024	77	25,7	
**Turno** ^F^			
Mañana (7:00–12:59)	83	36,6	
Tarde (13:00–18:59)	60	26,4	
Noche (19:00–06:59)	84	37,0	
**Clasificación de riesgo antes de la caída (Escala de Morse)** ^F^			<0,001
Alto	121	61,7	
Bajo	26	13,3	
Moderado	49	25,0	
**Clasificación de la caída** ^F^			<0,001
Grave	3	1,3	
Moderada	107	44,6	
Leve	130	54,2	
**Hospitalizaciones previas en el último año (2024)** ^F^			0,143
No	103	55,4	
Sí	83	44,6	
**Días de hospitalización en el período** ^D^, ^F^			–
Hasta siete días	45	26,6	
De ocho a 14 días	41	24,3	
De 15 a 21 días	37	21,9	
De 22 a 28 días	13	7,7	
De 29 a 35 días	15	8,9	
Más de 35 días	18	10,7	
**Área hospitalaria de la caída**			<0,001
Cirugía	78	27,9	
Medicina interna	106	37,9	
Urgencias para adultos	41	14,6	
Unidad de Cuidados Intensivos para adultos	10	3,6	
Pediatría	4	1,4	
Ambulatorio	4	1,4	
Alojamiento conjunto	11	3,9	
Otros	26	9,3	

Leyenda: A – porcentaje obtenido a partir del total de casos válidos; B – prueba de Chi-cuadrado de concordancia (compara las proporciones de las categorías de respuesta de una misma variable); D – variable con distribución asimétrica (prueba de Kolmogorov-Smirnov; p < 0,05); F: datos faltantes en el turno (73; 24,3 %), clasificación de riesgo antes de la caída (104; 34,7 %), clasificación de la caída (60; 20,0 %), hospitalizaciones previas (114; 38,0 %) y días de hospitalización (131; 43,7 %).

Fuente: Datos de la investigación.

Como se muestra en la Tabla 3, las caídas resultaron ser más frecuentes en pacientes hospitalizados (89,2 %), principalmente en la habitación (42,9 %) y en el baño (34,5 %), siendo más frecuentes las caídas desde la propia altura (54,9 %).

**Tabla 3:** Distribución absoluta y relativa de las variables relacionadas con el evento de caídas – Florianópolis, SC, Brasil, 2026.

Variables	Total (n = 300)^A^		Test X^2^ de adherencia Valor de p^B^
	n	%	
**Situación** ^F^			<0,001
Consultorio	5	1,7	
Atención de urgencia/emergencia	20	6,7	
Hospitalización	265	89,2	
Otros	7	2,4	
**Lugar del incidente** ^F^			<0,001
Baño	102	34,5	
Habitación	127	42,9	
Pasillo	25	8,4	
Otros	38	12,8	
No sabe informar	4	1,4	
**Característica** ^F^			<0,001
Silla de baño	15	5,1	
Cama	63	21,5	
Camillas	15	5,1	
Altura propia	161	54,9	
Silla de ruedas	9	3,1	
Sillones	6	2,0	
Otros	24	8,2	
**Factores de riesgo**			<0,001^E^
Edad	136	45,3	
Diagnóstico	182	60,7	
Medicamentos	50	16,7	
Factores cognitivos	65	21,7	
Cirugía/sedación/anestesia	26	8,7	
Otros	64	21,3	

Leyenda: A – porcentaje obtenido a partir del total de casos válidos; B – prueba de Chi-cuadrado de concordancia (compara las proporciones de las categorías de respuesta de una misma variable); E – prueba Q de Cochran; F – datos faltantes en situación (3; 1,0 %), lugar del incidente (4; 1,3 %) y característica (7; 2,3 %).

Fuente: Datos de la investigación.

Entre las posibles causas, se destacaron la falta de equilibrio (n = 111; 37,0 %), la pérdida de fuerza (n = 93; 31,0 %) y la desorientación (n = 89; 30 %). Solo el 14 % (n = 43) señaló la estructura del edificio y los materiales hospitalarios como posibles causas. La mayor parte de las notificaciones fue realizada por el equipo de enfermería (n = 259; 86,6 %). Las circunstancias que los profesionales notificantes asociaron con mayor frecuencia a las caídas incluyeron el estado clínico del paciente (n = 198; 66,0 %), la nutrición inadecuada (n = 144; 48,0 %) y la realización de múltiples procedimientos (n = 126; 42,0 %). Por otro lado, la falta de orientación para la prevención de caídas (n = 61; 20,3 %) y el tamaño del equipo (n = 31; 10,3 %) se señalaron con menor frecuencia como circunstancias relacionadas con el evento adverso.

En el análisis bivariado, la ocurrencia de caídas moderadas o graves se asoció con el sexo masculino (RP = 1,43; IC 95 %: 0,94–2,43; p = 0,069), con una edad más temprana (RP = 0,99; IC 95 %: 0,97–1,00; p = 0,093), a ser de raza no blanca (RP = 1,28; IC 95 %: 0,91–1,32; p = 0,086), a no vivir en una unión estable (RP = 1,66; IC 95 %: 0,85–2,77; p = 0,091) y a un menor nivel educativo. Entre las variables clínicas y relacionadas con las caídas, se destacaron la falta de equilibrio (RP = 0,80; IC 95 %: 0,63–1,01; p = 0,064), la desorientación (RP = 1,33; IC 95 %: 0,86–2,33; p = 0,214) y los factores estructurales (RP = 1,64; IC 95 %: 0,83–3,45; p = 0,187). Solo se observó una asociación estadísticamente significativa para las hospitalizaciones previas (RP = 0,72; IC 95 %: 0,43–0,91; p = 0,009), lo que se configuró como un factor de protección.

En los modelos intrabloc, se mantuvieron como predictores en el bloque sociodemográfico el color autodeclarado no blanco (RP = 2,46; IC 95 %: 1,12–5,41; p = 0,025) y la edad más joven (RP = 0,99; IC 95 %: 0,86–1,01; p = 0,099), con una varianza explicada por el color autodeclarado y la edad sobre los tipos de gravedad de la caída del 7,8 % (R^2^ de Nagelkerke = 0,078) y una precisión del 63,9 %. En el bloque clínico, se mantuvieron el hábito de consumir alcohol (RP = 1,55; IC 95 %: 0,92–3,26; p = 0,064), las enfermedades crónicas (RP = 1,76; IC 95 %: 1,10–3,88; p = 0,004), la realización de un procedimiento (RP = 1,35; IC 95 %: 0,95–2,34; p = 0,063) y las enfermedades del sistema circulatorio (RP = 2,85; IC 95 %: 1,19–6,44; p = 0,008), con un R^2^ = 0,156 y una precisión del 61,7 %. En el bloque relativo a las características de la caída, se destacaron el turno de la mañana (RP = 2,35; IC 95 %: 1,93–6,98; p = 0,027) y las hospitalizaciones previas (RP = 0,32; IC 95 %: 0,41–0,75; p = 0,009), con un R^2^ = 0,119 y una precisión del 60,7 %.

Como se muestra en la Tabla 4, en el modelo final, no hubo una reducción significativa de la varianza explicada (R^2^ ajustado: 0,522 frente a 0,497), de la asociación entre variables (0,389 frente a 0,327) ni de la precisión (74,4 % frente a 69,2 %), lo que indica que se mantuvo el poder predictivo con un menor número de variables. Mediante el método *backward conditional*, los principales predictores de caídas moderadas/graves fueron la duración de la hospitalización >14 días (RP = 7,21; IC 95 %: 1,68–26,47), el sexo masculino (RP = 2,89; IC 95 %: 1,15–7,40), la ocurrencia de una caída durante el turno de la mañana (RP = 2,54; IC 95 %: 1,17–6,73), presentar desorientación como posible causa del incidente (RP = 1,86; IC 95 %: 1,11–4,55), las enfermedades crónicas (RP = 1,36; IC del 95 %: 1,11–3,69) y que el motivo de la hospitalización fueran enfermedades del sistema circulatorio (RP = 1,58; IC del 95 %: 1,03–4,31), mientras que las hospitalizaciones previas mantuvieron un efecto protector (RP = 0,37; IC del 95 %: 0,22–0,94) frente a las caídas moderadas o graves.

**Tabla 4:** Modelo final de regresión de Poisson para los factores asociados a la ocurrencia de caídas moderadas/graves – Florianópolis, SC, Brasil, 2026.

Variables independientes estimadas para el modelo final	Valor de p	Razón de prevalencia para caídas moderadas/graves		
		RP	IC95%	
**Datos sociodemográficos**				
Hombres	0,020	2,885	1,146	7,400
**Clínicas**				
Enfermedades del sistema circulatorio	0,034	1,576	1,026	4,311
Enfermedades crónicas	0,027	1,357	1,105	3,689
**Específicas de las caídas**				
Turno nocturno	0,038	1,000		
Turno matutino	0,002	2,541	1,174	6,725
Turno vespertino	0,078	1,118	0,922	3,447
Hospitalizaciones previas en el último año (2024)	0,026	0,374	0,218	0,941
Período de hospitalización				
Hasta siete días	0,022			
De ocho a 14 días	0,440	3,211	0,166	62,175
De 15 a 21 días y más de 21 días	0,006	7,208	1,680	26,472
**Posibles causas del incidente**				
Desorientación	0,017	1,856	1,113	4,551
Constante	0,154	0,006		

Leyenda: RP: razón de prevalencia (estimada mediante el modelo de regresión de Poisson); IC95%: intervalo de confianza del 95 % para la razón de prevalencia; parámetros del modelo: Chi-cuadrado de la razón de verosimilitud (622,341; p = 0,564); proporción de varianza explicada (R^2^ ajustado = 0,497); grado de asociación entre las variables predictoras y la variable dependiente (Cox y Snell = 0,327); matriz de confusión (69,2 %).

Fuente: Datos de la investigación.

En cuanto a la capacidad predictiva del modelo final, el área bajo la curva ROC fue de 0,768 (IC95%: 0,681 – 0,812; p < 0,001) (Figura 1), lo que indica una capacidad de discriminación moderada por encima del azar (50,0%). Las estimaciones de sensibilidad y especificidad fueron de 74,6 % y 62,3 %, respectivamente. Por lo tanto, hay evidencia de que las clasificaciones no se predijeron por casualidad.

**Figura 1:** Curva ROC para el modelo logístico predictivo de caídas moderadas/graves - Florianópolis, SC, Brasil, 2026.

Fuente: Datos de la investigación.

## DISCUSIÓN

Los años con mayor concentración de caídas fueron 2024 (25,7 %) y 2019 (20,7 %). Entre esos períodos se produjo la pandemia de COVID-19, que exigió la reestructuración de los servicios de salud y la reasignación de recursos, lo que posiblemente dio lugar a un subregistro de eventos adversos sin que ello representara una reducción real en su ocurrencia. Se describieron hallazgos similares en un estudio multicéntrico italiano, que también evidenció variaciones anuales significativas en el número de caídas hospitalarias durante y después de la pandemia^(4)^.

En cuanto al sexo, se identificó un predominio masculino (55 %) y un riesgo aproximadamente dos veces mayor de sufrir caídas moderadas y graves (RP = 2,89). Este resultado difiere de los estudios que señalan un mayor riesgo en las mujeres^(16)^, y puede explicarse por las características del hospital estudiado, de la población atendida en el servicio de salud analizado y de las demás variables presentes en el estudio^(16)^.

Las enfermedades crónicas estaban presentes en el 86,2 % de la población y se asociaron significativamente con caídas moderadas y graves (RP = 1,36), al igual que las enfermedades del sistema circulatorio (RP = 1,58). Estos resultados concuerdan con la evidencia que relaciona comorbilidades, como la HAS, la diabetes, la insuficiencia cardíaca, el accidente cerebrovascular y la enfermedad hepática, con una mayor susceptibilidad a las caídas y a las lesiones, principalmente en adultos mayores^(17)^. El hospital analizado atiende a pacientes de complejidad media y alta del Sistema Único de Salud (SUS), y concentra casos con múltiples afecciones clínicas y procedimientos invasivos.

Aunque los períodos matutino (36,6 %) y nocturno (37,0 %) presentaron proporciones similares de caídas, el modelo predictivo identificó que el turno de la mañana se asoció con un riesgo 2,5 veces mayor de caídas moderadas y graves (RP = 2,54). Este hallazgo podría estar relacionado con la mayor concentración de actividades de atención en ese período, como baños, cambio de ropa de cama, exámenes y traslado de pacientes, tal como se describe en estudios internacionales^(18)^.

Las caídas ocurrieron predominantemente en pacientes hospitalizados (89,2 %), sobre todo en las habitaciones (42,9 %) y los baños (34,5 %). Estos ambientes se describen como lugares críticos en la literatura. Otro estudio también evidenció que las caídas ocurrieron principalmente en las habitaciones de hospitalización (64,2 %), seguidas de los baños (35,8 %) y los pasillos (10 %)^(5)^. Este hallazgo pone de manifiesto la necesidad de intervenciones estructurales, como barras de apoyo, pisos antideslizantes e iluminación adecuada, además de una vigilancia intensificada por parte del equipo de enfermería.

Los pacientes hospitalizados presentan una mayor susceptibilidad a las caídas intrahospitalarias debido a múltiples factores identificados por los profesionales de la salud, como el estado clínico del paciente (66 %, la nutrición inadecuada (48 %), la edad avanzada (45,3 %), la falta de equilibrio (37 %), la pérdida de fuerza (31 %) y el diagnóstico clínico (60,7 %). Desde esta perspectiva, se observó que los pacientes con desorientación como posible causa de la caída presentaban una mayor probabilidad de sufrir una caída moderada o grave (PR = 1,86). Resultados similares evidenciaron que, además de otros factores, la desorientación fue un factor de riesgo medio y alto (OR de 2,40 y 12,54, respectivamente) para las caídas^(19)^.

La caída desde la propia altura fue el mecanismo más frecuente en este estudio (54,9 %), asociada a los factores intrínsecos descritos en el párrafo anterior. Estos hallazgos coinciden con una revisión sistemática reciente que identificó la alteración de la marcha y del equilibrio como un fuerte predictor de caídas (OR = 2,11). De manera similar, la desorientación presente en el 30 % de los pacientes de este estudio, asociada a caídas con desenlaces moderados y graves (RP = 1,86), concuerda con la evidencia internacional que señala el deterioro cognitivo y el delirio como factores críticos para las caídas (OR = 2,30)^(20)^.

El motivo de hospitalización más frecuente fue el grupo de enfermedades del aparato digestivo (20,6 %), seguido de procedimientos quirúrgicos (18,2 %) y enfermedades circulatorias (14 %). Aunque las enfermedades del aparato circulatorio no se destacaron en el análisis descriptivo, se observaron razones de prevalencia significativas en el conjunto de variables incluidas en el modelo predictivo. Las afecciones identificadas con mayor frecuencia en este grupo fueron insuficiencia cardíaca, enfermedad arterial obturativa periférica, trombosis venosa profunda, accidente cerebrovascular, isquemia y aneurisma. A pesar de que la variable relativa a los pacientes sometidos a procedimientos no se mantuvo en el modelo final, la amputación se destacó como uno de los principales procedimientos registrados.

En este contexto, los pacientes con enfermedades circulatorias descompensadas pueden presentar un mayor riesgo de amputación de extremidades, un evento que tiene un impacto sustancial en la vida del individuo, ya que compromete la calidad de vida, reduce la movilidad y la participación en actividades sociales, además de estar asociado a síntomas de ansiedad, depresión e inseguridad ante las nuevas condiciones impuestas^(21,22)^. Además, otro estudio destaca la mayor prevalencia de caídas tardías (más de diez días) en pacientes con afectación de las extremidades, en comparación con aquellos que no presentan estos síntomas (2,58; IC 95 % = 1,40–4,76)^(23)^.

Los antecedentes de hospitalización en el último año (2024) demostraron ser un factor de protección frente a las caídas con consecuencias moderadas y graves (RP = 0,37). Una posible explicación de este hallazgo es que dichos pacientes ya contaban con conocimientos previos sobre el riesgo de caídas, además de una mayor familiaridad con la rutina hospitalaria y con las medidas preventivas implementadas por el equipo de enfermería. Este hallazgo puede reforzarse por el hecho de que solo el 20,3 % de los profesionales notificadores identificaron la falta de orientación para la prevención de caídas como una circunstancia potencialmente asociada a la ocurrencia del evento^(24)^.

La estancia hospitalaria prolongada está fuertemente asociada con peores resultados clínicos y un mayor riesgo de eventos adversos. En el presente estudio, la estancia superior a 14 días fue el factor de mayor impacto para la clasificación de caídas moderadas y graves (RP = 7,21). Otro autor describió hallazgos similares, al observar un promedio de 14,2 días de hospitalización en el grupo con caídas y de 4,7 días en el grupo sin caídas, lo que indica que una mayor estancia hospitalaria está asociada a la ocurrencia del evento^(25)^.

Aunque la estancia hospitalaria prolongada se asocia con un mayor riesgo de caídas, no debe entenderse únicamente como un factor aislado, sino como un reflejo de la complejidad clínica del paciente, lo que incluye una mayor fragilidad física, como la pérdida de equilibrio y de fuerza (variables también identificadas en este estudio), además de una mayor exposición a procedimientos médicos y el agravamiento de afecciones preexistentes. Estos aspectos hacen que el paciente sea más susceptible a sufrir caídas con consecuencias más graves^(26)^.

Además, especialmente en personas mayores, la prolongación de la hospitalización puede favorecer la pérdida funcional adquirida durante la estancia hospitalaria, lo que incluye sarcopenia, reducción de la capacidad funcional y deterioro del equilibrio postural. Este descondicionamiento se asocia con un mayor riesgo de caídas, fracturas, delirio, infecciones hospitalarias, pérdida de independencia tras el alta y aumento de la duración de la hospitalización. Por lo tanto, se requieren medidas preventivas desde el momento del ingreso hospitalario^(27)^.

La incidencia de caídas, predominantemente en pacientes clasificados como de alto riesgo (61,7 %), corrobora la capacidad predictiva de la Escala de Morse. Sin embargo, este hallazgo demuestra que la estrategia de prevención basada únicamente en el puntaje total de la escala puede ser insuficiente, por lo que es necesario pasar a intervenciones basadas en los componentes individuales del riesgo, tal como lo sugiere un amplio estudio multicéntrico realizado en 72 hospitales. Este hallazgo refuerza la necesidad de reevaluaciones dinámicas, ya que las medidas preventivas estáticas implementadas al momento de la admisión podrían no seguir el ritmo de los cambios clínicos del paciente a lo largo de la hospitalización^(28)^.

Aunque solo el 14 % (n = 43) de los profesionales que notificaron el evento señalaron la infraestructura física y los materiales hospitalarios como posibles causas de la caída, y el 10 % (n = 31) relacionaron el evento con la dotación de personal, es posible que estos factores estén subestimados. Las cuestiones organizativas tienden a ser menos reconocidas durante el análisis del evento adverso, ya que el equipo de atención suele centrar su atención en el estado clínico del paciente y en los procesos inmediatos de atención^(29)^. Sin embargo, incluso cuando los profesionales conocen los protocolos de prevención, las limitaciones estructurales y las insuficiencias en la dotación de personal pueden dificultar la implementación efectiva de estas medidas preventivas^(29)^.

El predominio del personal de enfermería como categoría notificadora (86,6 %) refleja su papel central en la vigilancia y la seguridad del paciente, debido a su contacto continuo con los pacientes hospitalizados. Sin embargo, es fundamental fortalecer la participación multiprofesional, incentivando que todas las categorías participen activamente en el proceso de notificación y en la implementación de estrategias preventivas^(30)^.

La investigación se llevó a cabo en un único hospital general que brinda atención exclusivamente a través del SUS, lo que refleja la realidad institucional y asistencial de ese contexto específico. Por lo tanto, los resultados deben interpretarse con cautela en cuanto a su generalización a otros escenarios, especialmente aquellos con diferentes perfiles de financiamiento, gestión o complejidad asistencial. Sin embargo, las características del hospital estudiado pueden hacer que los hallazgos sean relevantes y potencialmente aplicables a otras instituciones con un perfil similar.

## CONCLUSIÓN

El estudio permitió caracterizar a los pacientes afectados por caídas hospitalarias. En este contexto, predominó el sexo masculino, con una edad promedio de 56 años, siendo el grupo de edad de 50 a 59 años el más representativo. La mayoría de los pacientes que sufrieron una caída (86,2 %) presentaba enfermedades crónicas. Las caídas ocurrieron principalmente entre pacientes hospitalizados. Entre los motivos de hospitalización, se destacaron las enfermedades del sistema circulatorio y respiratorio, con predominio de pacientes previamente clasificados como de alto riesgo de caídas. La mayoría de las caídas (54,9 %) fue desde la propia altura.

Además, se identificaron como factores predictivos de caídas moderadas y graves en pacientes hospitalizados el sexo masculino, una mayor duración de la hospitalización, las enfermedades crónicas, las enfermedades del sistema circulatorio, la desorientación y el turno matutino. Haber tenido hospitalizaciones previas en el último año fue un factor de protección frente a las caídas con ese desenlace. El modelo final presentó una capacidad discriminatoria moderada, lo que refuerza su utilidad como herramienta para apoyar la práctica clínica y orientar las estrategias de prevención dirigidas a los grupos de mayor riesgo.

Los hallazgos resaltan la importancia de invertir en medidas preventivas específicas en el hospital estudiado, como la intensificación de la vigilancia durante los períodos matutinos, la evaluación rigurosa del estado clínico de los pacientes con comorbilidades y la adaptación del entorno físico de las habitaciones y los baños. El estudio contribuye al avance de la seguridad del paciente en el contexto hospitalario brasileño y señala la necesidad de nuevas investigaciones multicéntricas que validen y amplíen el modelo predictivo aquí propuesto.

## Data Availability

Todo el conjunto de datos que respalda los resultados de este estudio se publicó en el propio artículo.
